# Genetic ancestry and diagnostic yield of exome sequencing in a diverse population

**DOI:** 10.1038/s41525-023-00385-6

**Published:** 2024-01-03

**Authors:** Yusuph Mavura, Nuriye Sahin-Hodoglugil, Ugur Hodoglugil, Mark Kvale, Pierre-Marie Martin, Jessica Van Ziffle, W. Patrick Devine, Sara L. Ackerman, Barbara A. Koenig, Pui-Yan Kwok, Mary E. Norton, Anne Slavotinek, Neil Risch

**Affiliations:** 1 Institute for Human Genetics, University of California San Francisco, San Francisco, CA USA; 2 Department of Epidemiology & Biostatistics, University of California San Francisco, San Francisco, CA USA; 3 Department of Pathology, University of California San Francisco, San Francisco, CA USA; 4 Institute for Health & Aging, School of Nursing, University of California San Francisco, San Francisco, CA USA; 5 Department of Social & Behavioral Sciences, School of Nursing, University of California San Francisco, San Francisco, CA USA; 6 Program in Bioethics, University of California San Francisco, San Francisco, CA USA; 7 Cardiovascular Research Institute and Department of Dermatology, University of California San Francisco, San Francisco, CA USA; 8 Division of Maternal Fetal Medicine, Department of Obstetrics, Gynecology, and Reproductive Sciences, University of California, San Francisco, San Francisco, CA USA; 9 Department of Pediatrics, University of California, San Francisco, San Francisco, CA USA

**Keywords:** Genetic testing, Genetics research

## Abstract

It has been suggested that diagnostic yield (DY) from Exome Sequencing (ES) may be lower among patients with non-European ancestries than those with European ancestry. We examined the association of DY with estimated continental/subcontinental genetic ancestry in a racially/ethnically diverse pediatric and prenatal clinical cohort. Cases (*N* = 845) with suspected genetic disorders underwent ES for diagnosis. Continental/subcontinental genetic ancestry proportions were estimated from the ES data. We compared the distribution of genetic ancestries in positive, negative, and inconclusive cases by Kolmogorov–Smirnov tests and linear associations of ancestry with DY by Cochran-Armitage trend tests. We observed no reduction in overall DY associated with any genetic ancestry (African, Native American, East Asian, European, Middle Eastern, South Asian). However, we observed a relative increase in proportion of autosomal recessive homozygous inheritance versus other inheritance patterns associated with Middle Eastern and South Asian ancestry, due to consanguinity. In this empirical study of ES for undiagnosed pediatric and prenatal genetic conditions, genetic ancestry was not associated with the likelihood of a positive diagnosis, supporting the equitable use of ES in diagnosis of previously undiagnosed but potentially Mendelian disorders across all ancestral populations.

## Introduction

Advances in exome sequencing (ES) technology have led to use of ES in establishing molecular diagnoses for Mendelian diseases in children and adults. This has prompted recommendations for ES as the first line genetic test for certain clinical indications such as neurodevelopmental disorders^1^. The probability of a positive case classification from ES (diagnostic yield) may differ due to factors such as: number of parents sequenced with proband, parental age, variant of uncertain significance (VUS) calling threshold, consanguinity, clinical indication or phenotype presentation, sex and age of proband, genetic ancestry, or a combination of these factors. Most of the studies on ES diagnostic yield have been conducted in predominantly European ancestry populations^2^.

Relatively little is known about the diagnostic yield (DY) from ES in individuals with ancestry such as African, East Asian, South/Central Asian, Middle Eastern, Native American, as well as ancestrally admixed individuals^3^. Genetic variant data from individuals with non-European ancestry is less well represented in genetic and genomic databases^2^, and it has been suggested that DY may be lower in those with non-European ancestry. Some have found higher rates of VUSs in individuals with African, and Native American compared to those of European ancestry^4,5^, which suggests the potential for reduced diagnostic yield in non-European ancestry populations.

To investigate this question in the context of ES for rare undiagnosed but suspected Mendelian disorders, we analyzed the association of diagnostic yield with estimated global genetic ancestry in an ancestrally diverse cohort of pediatric and prenatal cases who underwent ES, and how it relates to the self-identified race/ethnicity of the parents of the cases.

Our analysis was based in the Program in Pediatric and Prenatal Genomic Sequencing (P^3^EGS) cohort at the University of California, San Francisco (UCSF), which is part of the Clinical Sequencing Evidence-generating Research (CSER) consortium^6^. Cases in the P^3^EGS cohort had a wide range of clinical indications for ES, and was ancestrally diverse, with 70% of parents providing race/ethnicity information self-identifying as non-white^7^.

The association between diagnostic yield and important factors other than genetic ancestry has been reported previously, in a separate but related study, using the same cohort^7^. This work extends the results from that study, by specifically investigating genetic ancestry estimated from sequence data in relation to diagnostic yield.

## Results

### Participant demographics and exome sequencing

A total of 845 (529 pediatric, 316 prenatal) cases and their available biological parents were enrolled in the study primarily at one of five sites in the San Francisco Bay area and Central Valley of California (2 pediatric and 58 prenatal families were referred from outside California). Participants in the cohort had a wide range of clinical indications for ES^7^. There were more male (54.8% pediatric, 54.1% prenatal) than female cases in the cohort. Overall, 16.3% of pediatric cases were less than a year old, and 76.6% were 10 years or younger at enrollment. The median maternal and paternal age at proband conception in the pediatric cohort was 28.2 and 32.2 years, respectively. Among prenatal patients, the mean gestational age at enrollment was 23.5 weeks. The median maternal and paternal age at proband conception in the prenatal cohort was 33.1 and 35.0 years, respectively.

All 845 cases received ES. Among pediatric cases, ES was done on both parents in 337 cases (trio, quad ES), a single parent on 111 cases (duo ES), and neither parent of 81 cases. Among prenatal cases, ES was done on both parents of 262 cases (trio, quad, quint ES), one parent of 16 cases (duo ES), and neither parent of 38 cases, yielding a total of 1325 parents with ES data.

See ref. ^7^ for more details on the individuals studied and their demographics.

### Race/ethnicity and genetic ancestry of P^3^EGS participants

The parents of probands in the P^3^EGS cohort were racially and ethnically diverse. Among parents of pediatric probands, 40.7% were Latino(a), 18.6% white/European, 4.7% East Asian, 3.9% African American or Black, 2.6% Central Asian, 2.6% South Asian, 2.3% Middle Eastern, 1.1% Native American, 0.9% Pacific Islander, 7.2% multiethnic, and 15.4% missing. Among prenatal probands’ parents, the race/ethnicity breakdown was 36.4% white/European, 15.5% Latino(a), 9.0% East Asian, 5.4% South Asian, 0.9% African American or Black, 9.2% multiethnic, and 22.0% missing (Table 1).

**Table 1:** Distribution of self-identified race/ethnicity of parents of pediatric and prenatal cases in the P^3^EGS cohort.

	Pediatric		Prenatal	
Race/ethnicity categories	*N* (% of maternal race/ethnicity total)	*N* (% of paternal race/ethnicity total)	*N* (% of maternal race/ethnicity total)	*N* (% of paternal race/ethnicity total)
African American or Black (AF)	21 (3.97%)	20 (3.78%)	3 (0.95%)	3 (0.95%)
Central Asian (CA)	14 (2.65%)	14 (2.65%)	0 (0%)	0 (0%)
East Asian (EA)	28 (5.29%)	22 (4.16%)	31 (9.81%)	26 (8.23%)
white/European (EU)	96 (18.15%)	101 (19.09%)	115 (36.39%)	115 (36.39%)
Latino(a) (LT)	228 (43.1%)	203 (38.37%)	49 (15.51%)	49 (15.51%)
Middle Eastern (ME)	12 (2.27%)	12 (2.27%)	5 (1.58%)	5 (1.58%)
Native American (NAT)	6 (1.13%)	6 (1.13%)	0 (0%)	0 (0%)
Pacific Islander (PI)	4 (0.76%)	5 (0.95%)	0 (0%)	0 (0%)
South Asian (SA)	14 (2.65%)	13 (2.46%)	18 (5.7%)	16 (5.06%)
AF, EU	6 (1.13%)	2 (0.38%)	2 (0.63%)	3 (0.95%)
EA, EU	4 (0.76%)	2 (0.38%)	6 (1.9%)	1 (0.32%)
EU, LT	9 (1.7%)	6 (1.13%)	8 (2.53%)	14 (4.43%)
EU, NAT	8 (1.51%)	11 (2.08%)	4 (1.27%)	3 (0.95%)
Other 2 combination race/ethnicities	8 (1.51%)	8 (1.51%)	6 (1.9%)	2 (0.63%)
3 or More race/ethnicities	7 (1.32%)	5 (0.95%)	6 (1.9%)	3 (0.95%)
Missing (unknown/none of these fully describe me/prefer not to answer)	64 (12.1%)	99 (18.71%)	63 (19.94%)	76 (24.05%)
Total	529 (100%)	529 (100%)	316 (100%)	316 (100%)

Results of PC analysis are given in Supplementary Figs. 1–3. The first 6 PCs depict African, European, East Asian, Native American, South Asian, Middle Eastern and Pacific Islander genetic ancestries. The P^3^EGS cases reflect all these ancestries, with the largest components being European, Native American, and East Asian.

**Fig. 1:**
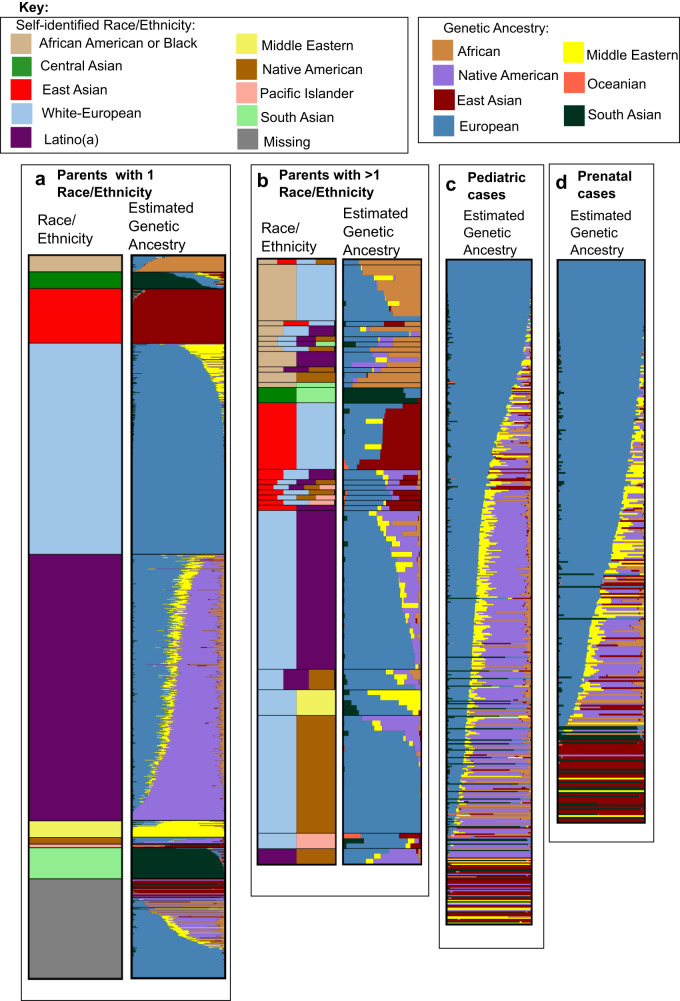
Correspondence between estimated genetic ancestry proportions and self-reported race/ethnicity of parents of P^3^EGS cases; and estimated genetic ancestry of pediatric and prenatal cases. **a** Correspondence between race/ethnicity and estimated global genetic ancestry admixture proportions in parents with 1 reported race/ethnicity. **b** Correspondence between race/ethnicity and estimated global genetic ancestry admixture proportions in parents with more than 1 reported race/ethnicity. **c** estimated global genetic ancestry admixture proportions in pediatric cases. **d** estimated global genetic ancestry admixture proportions in prenatal cases. Each horizontal bar in the “Estimated Genetic ancestry column” represents one parent or case, and the “Race/Ethnicity” column corresponds to the self-reported race/ethnicity of the parent. Genetic ancestry proportions/percentages were estimated from exome sequencing data using Admixture software with unrelated Human Genome Diversity Panel (HGDP) samples from gnomAD as reference samples/populations.

The correspondence between self-identified race/ethnicity and estimated individual genetic ancestry proportions of the 1325 exome sequenced parents is visualized in Fig. 1. This includes those whose race/ethnicity information was missing. As shown previously^8^, for those reporting a single race/ethnicity there is a high correspondence between genetic ancestry and self-reported race/ethnicity (Fig. 1a). For example, those reporting East Asian race/ethnicity have near 100% East Asian genetic ancestry; the same is true for those reporting South Asian, white/European, and Middle Eastern race/ethnicity. Those reporting African American or Black race/ethnicity have admixed African and European genetic ancestry, while Latino(a) participants have primarily Native American and European genetic ancestry, with a modest contribution of African and Middle Eastern ancestry. The genetic ancestry of Central Asians appears to be intermediate between South Asian and European/Middle Eastern. The genetic ancestry distribution of those with missing race/ethnicity appears quite comparable to the overall distribution of those with information, reflecting largely European genetic ancestry, mixed European/Native American genetic ancestry, East Asian, South Asian and African genetic ancestry. Parents who reported more than 1 race/ethnicity had a higher level of genetic admixture compared to those who reported only 1, and again there is a high correspondence between the self-reported race/ethnicities and genetic admixture for these participants (Fig. 1b). The single exception is for those reporting Native American and white/European race/ethnicity. The majority of such participants have only European genetic ancestry, while the remainder are admixed European with a modest to moderate amount of Native American genetic ancestry. This observation is comparable to what has been reported previously^8^. The average genetic ancestry proportions in the pediatric cases were: 41.6% European, 28.9% Native American, 7.2% East Asian, 8.6% Middle Eastern, 7.3% African, and 6.2% South Asian. The average genetic ancestry proportions in the prenatal cases were: 56.8% European, 10.6% Native American, 12.9% East Asian, 7.8% Middle Eastern, 4.5% African, and 7.2% South Asian. Combined, the estimated genetic ancestry proportions (mean, standard deviation) were: 47.3% (33.9%) European, 22.1% (27.6%) Native American, 9.3% (25.6%) East Asian, 8.3% (14.5%) Middle Eastern, 6.2% (16.5%) African, and 6.6% (21.3%) South Asian. The average estimated Oceanian genetic ancestry was less than 1% in both pediatric and prenatal cases, so we did not include it in subsequent analyses.

### Genetic ancestry and diagnostic yield

The diagnostic yield was significantly higher in pediatric compared to prenatal cases^7^. Overall, out of 529 pediatric probands, 141 (26.7%) had a positive (definitive + probable positive) case outcome and 73 (13.8%) had an inconclusive case outcome, while among 316 prenatal probands, 60 (19%) had a positive case outcome and 19 (6%) had an inconclusive case outcome.

The majority of the positive cases had an AD mode of inheritance: 70% and 65% in the pediatric and prenatal arms of the study, respectively, compared to 18% and 25%, respectively, that had AR inheritance. Compared to the positive cases, the inconclusive cases had a lower percentage that were of AD inheritance (41.1% pediatric, 60% prenatal) and a higher proportion of AR inheritance (45% pediatric, 30% prenatal).

**Fig. 2:**
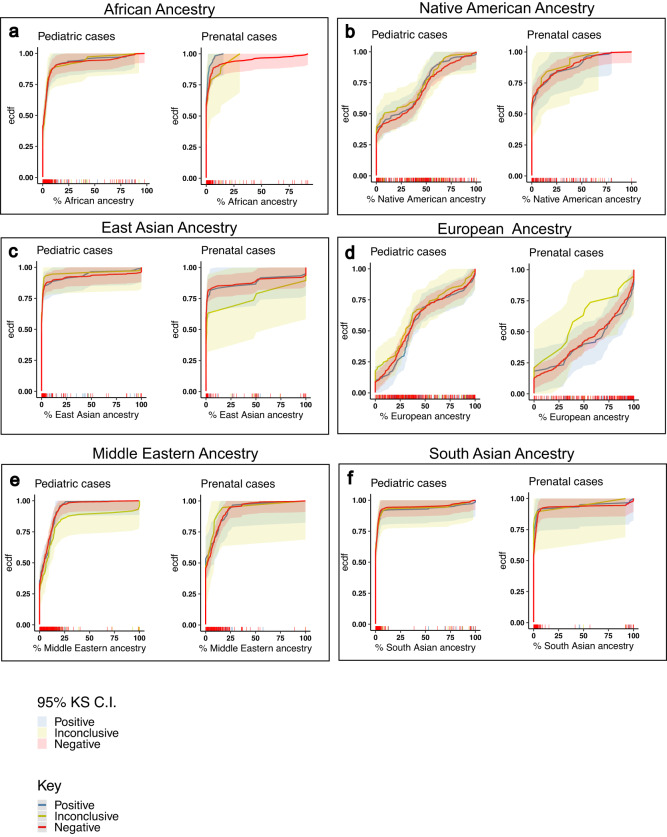
Empirical cumulative distribution functions (ECDF) and their corresponding 95% confidence interval (C.I.) bands of estimated genetic ancestries stratified by case outcome—positive, inconclusive, and negative, and by pediatric or prenatal cases. **a** ECDF for African ancestry. **b** ECDF for Native American ancestry. **c** ECDF for East Asian ancestry. **d** ECDF for European ancestry. **e** ECDF for Middle Eastern ancestry. **f** ECDF for South Asian ancestry. There was no statistically significant difference between ECDFs in negative and positive cases, or negative and inconclusive cases in any of the genetic ancestries. Statistics were performed using Kolmogorov–Smirnov (KS) test. The short vertical lines on the *x* axis represent cases, ordered by their % genetic ancestries, and colored by outcomes as seen in the legend/key. KS 95% C.I. bands for ECDFs were calculated using Dvoretzky–Kiefer–Wolfowitz (DKW) inequality.

For each of the six genetic ancestries, there was no statistically significant difference in genetic ancestry distributions between positive, negative, and inconclusive outcomes in both pediatric and prenatal cases (Fig. 2): *P* values from Kolmogorov–Smirnov tests comparing genetic ancestry distributions in positive vs negative and inconclusive vs negative cases within each genetic ancestry group were all greater than 0.1 and not statistically significant.

The distribution of estimated genetic ancestries of probands was observed to be both continuous and discrete in the different genetic ancestry groups. For Native American and European ancestries, the estimated genetic ancestries were more continuous, and for East Asian, South Asian, African, Middle Eastern ancestries, the distribution of estimated genetic ancestries were more discrete. A clear example can be seen in Fig. 2, in the estimated East Asian ancestry panel, in which the groups of cases are clumped around the 0, 25, 50, 75, 100% estimated East Asian ancestry mark, with few cases in between. These percentages represent the number of grandparents from that ancestral population (0, 1, 2, 3 or 4). For that reason, genetic ancestry bins, containing frequency of cases (and their diagnostic yield) within estimated genetic ancestry ranges were made to best capture variation in diagnostic yield in estimated genetic ancestries (see 'Methods').

By the Cochran-Armitage test, there was no significant association between any genetic ancestry and diagnostic yield (Table 2). However, Middle Eastern genetic ancestry was significantly positively associated with an inconclusive outcome among pediatric probands, largely driven by the 78% (7/9) inconclusive rate in the highest Middle Eastern ancestry bin, compared to 11% (1/9) among negative cases (Table 2). However, this association was not observed in prenatal cases (Table 2).

**Table 2:** Number and diagnostic yield (in parentheses) of pediatric and prenatal cases by genetic ancestry bins, stratified by mode of inheritance, and Cochran-Armitage (C-A) test Z-statistics for trend tests of positive versus negative and inconclusive versus negative cases.

				Number of Cases & (%) in Each Bin					
Ancestry	Case definition	Mode of inheritance	*N*	0–12.5%	12.5–37.5%	37.5–62.5%	62.5–87.5%	87.5–100%	C-A test Z-statistic
Pediatric cases									
African	Positive	Autosomal dominant	99	87 (0.18)	5 (0.25)	2 (0.2)	4 (0.22)	1 (0.17)	0.23
		Autosomal recessive	25	22 (0.05)	2 (0.1)	1 (0.1)	0 (0)	0 (0)	−0.46
		X-linked	17	17 (0.04)	0 (0)	0 (0)	0 (0)	0 (0)	−1.23
		**All**	**141**	**126** **(0.27)**	**7** **(0.35)**	**3** **(0.3)**	**4** **(0.22)**	**1** **(0.17)**	−0.34
	Inconclusive	Autosomal dominant	30	25 (0.05)	3 (0.15)	2 (0.2)	0 (0)	0 (0)	0.01
		Autosomal recessive	32	30 (0.06)	0 (0)	0 (0)	1 (0.06)	1 (0.17)	−0.09
		X-linked	11	10 (0.02)	0 (0)	1 (0.1)	0 (0)	0 (0)	−0.21
		**All**	**73**	**65** **(0.14)**	**3** **(0.15)**	**3** **(0.3)**	**1** **(0.06)**	**1** **(0.17)**	−0.13
	Negative	Negative	315	284 (0.6)	10 (0.5)	4 (0.4)	13 (0.72)	4 (0.67)	
	Total		529	475	20	10	18	6	
Native American	Positive	Autosomal dominant	99	42 0.18)	15 (0.22)	29 (0.19)	8 (0.15)	5 (0.23)	−0.17
		Autosomal recessive	25	15 (0.06)	2 (0.03)	7 (0.05)	0 (0)	1 (0.05)	−1.59
		X-linked	17	7 (0.03)	1 (0.01)	8 (0.05)	1 (0.02)	0 (0)	−0.10
		**All**	**141**	**64** **(0.27)**	**18** **(0.27)**	**44** **(0.29)**	**9** **(0.17)**	**6** **(0.27)**	−0.75
	Inconclusive	Autosomal dominant	30	16 (0.07)	4 (0.06)	6 (0.04)	4 (0.08)	0 (0)	−1.16
		Autosomal recessive	32	16 (0.07)	2 (0.03)	10 (0.07)	2 (0.04)	2 (0.09)	−0.35
		X-linked	11	5 (0.02)	0 (0)	5 (0.03)	1 (0.02)	0 (0)	−0.06
		**All**	**73**	**37** **(0.16)**	**6** **(0.09)**	**21** **(0.14)**	**7** **(0.13)**	**2** **(0.09)**	−0.95
	Negative	Negative	315	136 (0.57)	43 (0.64)	85 (0.57)	37 (0.7)	14 (0.64)	
	Total		529	237	67	150	53	22	
East Asian	Positive	Autosomal dominant	99	89 (0.19)	3 (0.23)	6 (0.55)	0 (0)	1 (0.04)	−0.97
		Autosomal recessive	25	22 (0.05)	1 (0.08)	0 (0)	0 (0)	2 (0.08)	0.34
		X-linked	17	14 (0.03)	1 (0.08)	0 (0)	0 (0)	2 (0.08)	0.97
		**All**	**141**	**125** **(0.26)**	**5** **(0.38)**	**6** **(0.55)**	**0** **(0)**	**5** **(0.21)**	−0.32
	Inconclusive	Autosomal dominant	30	28 (0.06)	0 (0)	1 (0.09)	0 (0)	1 (0.04)	−0.51
		Autosomal recessive	32	30 (0.06)	0 (0)	0 (0)	0 (0)	2 (0.08)	−0.24
		X-linked	11	11 (0.02)	0 (0)	0 (0)	0 (0)	0 (0)	−1.01
		**All**	**73**	**69** **(0.14)**	**0** **(0)**	**1** **(0.09)**	**0** **(0)**	**3** **(0.13)**	−0.83
	Negative	Negative	315	283 (0.59)	8 (0.62)	4 (0.36)	4 (1)	16 (0.67)	
	Total		529	477	13	11	4	24	
European	Positive	Autosomal dominant	99	11 (0.11)	39 (0.2)	24 (0.24)	8 (0.17)	17 (0.2)	0.86
		Autosomal recessive	25	7 (0.07)	10 (0.05)	3 (0.03)	0 (0)	5 (0.06)	−0.87
		X-linked	17	2 (0.02)	9 (0.05)	0 (0)	2 (0.04)	4 (0.05)	0.44
		**All**	**141**	**20** **(0.21)**	**58** **(0.29)**	**27** **(0.27)**	**10** **(0.22)**	**26** **(0.3)**	0.49
	Inconclusive	Autosomal dominant	30	5 (0.05)	9 (0.05)	6 (0.06)	5 (0.11)	5 (0.06)	0.74
		Autosomal recessive	32	12 (0.12)	15 (0.08)	1 (0.01)	2 (0.04)	2 (0.02)	−2.91
		X-linked	11	2 (0.02)	3 (0.02)	2 (0.02)	1 (0.02)	3 (0.03)	0.79
		**All**	**73**	**19** **(0.2)**	**27** **(0.14)**	**9** **(0.09)**	**8** **(0.17)**	**10** **(0.11)**	−1.08
	Negative	Negative	315	58 (0.6)	115 (0.58)	63 (0.64)	28 (0.61)	51 (0.59)	
	Total		529	97	200	99	46	87	
Middle Eastern	Positive	Autosomal dominant	99	77 (0.19)	22 (0.19)	0 (0)	0 (0)	0 (0)	−0.55
		Autosomal recessive	25	19 (0.05)	5 (0.04)	0 (0)	0 (0)	1 (0.11)	0.92
		X-linked	17	12 (0.03)	5 (0.04)	0 (0)	0 (0)	0 (0)	0.31
		**All**	**141**	**108** **(0.27)**	**32** **(0.28)**	**0** **(0)**	**0** **(0)**	**1** **(0.11)**	0.03
	Inconclusive	Autosomal dominant	30	21 (0.05)	5 (0.04)	1 (0.5)	1 (0.33)	2 (0.22)	2.97
		Autosomal recessive	32	20 (0.05)	8 (0.07)	0 (0)	0 (0)	4 (0.44)	4.11*
		X-linked	11	8 (0.02)	2 (0.02)	0 (0)	0 (0)	1 (0.11)	1.70
		**All**	**73**	**49** **(0.12)**	**15** **(0.13)**	**1** **(0.5)**	**1** **(0.33)**	**7** **(0.78)**	4.29*
	Negative	Negative	315	243 (0.61)	68 (0.59)	1 (0.5)	2 (0.67)	1 (0.11)	
	Total		529	400	115	2	3	9	
South Asian	Positive	Autosomal dominant	99	92 (0.19)	1 (0.5)	0 (0)	3 (0.19)	3 (0.23)	0.43
		Autosomal recessive	25	20 (0.04)	0 (0)	2 (0.4)	1 (0.06)	2 (0.15)	2.47
		X-linked	17	17 (0.03)	0 (0)	0 (0)	0 (0)	0 (0)	−1.00
		**All**	**141**	**129** **(0.26)**	**1** **(0.5)**	**2** **(0.4)**	**4** **(0.25)**	**5** **(0.38)**	0.96
	Inconclusive	Autosomal dominant	30	30 (0.06)	0 (0)	0 (0)	0 (0)	0 (0)	−1.33
		Autosomal recessive	32	27 (0.05)	0 (0)	0 (0)	3 (0.19)	2 (0.15)	2.28
		X-linked	11	11 (0.02)	0 (0)	0 (0)	0 (0)	0 (0)	−0.81
		**All**	**73**	**68** **(0.14)**	**0** **(0)**	**0** **(0)**	**3** **(0.19)**	**2** **(0.15)**	0.48
	Negative	Negative	315	296 (0.6)	1 (0.5)	3 (0.6)	9 (0.56)	6 (0.46)	
	Total		529	493	2	5	16	13	

Prenatal cases									
African	Positive	Autosomal dominant	39	38 (0.13)	1 (0.07)	0 (0)	0 (0)	0 (0)	−1.46
		Autosomal recessive	15	15 (0.05)	0 (0)	0 (0)	0 (0)	0 (0)	−1.05
		X-linked	6	6 (0.02)	0 (0)	0 (0)	0 (0)	0 (0)	−0.67
		**All**	**60**	**59** **(0.2)**	**1** **(0.07)**	**0** **(0)**	**0** **(0)**	**0** **(0)**	−1.90
	Inconclusive	Autosomal dominant	11	7 (0.02)	4 (0.29)	0 (0)	0 (0)	0 (0)	0.86
		Autosomal recessive	6	6 (0.02)	0 (0)	0 (0)	0 (0)	0 (0)	−0.67
		X-linked	2	2 (0.01)	0 (0)	0 (0)	0 (0)	0 (0)	−0.39
		**All**	**19**	**15** **(0.05)**	**4** **(0.29)**	**0** **(0)**	**0** **(0)**	**0** **(0)**	0.16
	Negative	Negative	237	216 (0.74)	9 (0.64)	4 (1)	5 (1)	3 (1)	
	Total		316	290	14	4	5	3	
Native American	Positive	Autosomal dominant	39	27 (0.11)	6 (0.17)	5 (0.16)	1 (0.1)	0 (0)	0.56
		Autosomal recessive	15	12 (0.05)	1 (0.03)	2 (0.06)	0 (0)	0 (0)	−0.36
		X-linked	6	5 (0.02)	0 (0)	0 (0)	1 (0.1)	0 (0)	0.27
		**All**	**60**	**44** **(0.18)**	**7** **(0.19)**	**7** **(0.23)**	**2** **(0.2)**	**0** **(0)**	0.35
	Inconclusive	Autosomal dominant	11	8 (0.03)	1 (0.03)	2 (0.06)	0 (0)	0 (0)	0.18
		Autosomal recessive	6	5 (0.02)	0 (0)	0 (0)	1 (0.1)	0 (0)	0.27
		X-linked	2	2 (0.01)	0 (0)	0 (0)	0 (0)	0 (0)	−0.71
		**All**	**19**	**15** **(0.06)**	**1** **(0.03)**	**2** **(0.06)**	**1** **(0.1)**	**0** **(0)**	0.06
	Negative	Negative	237	179 (0.75)	28 (0.78)	22 (0.71)	7 (0.7)	1 (1)	
	Total		316	238	36	31	10	1	
East Asian	Positive	Autosomal dominant	39	32 (0.12)	1 (0.2)	3 (0.19)	0 (0)	3 (0.11)	0.13
		Autosomal recessive	15	13 (0.05)	0 (0)	0 (0)	0 (0)	2 (0.07)	0.23
		X-linked	6	5 (0.02)	0 (0)	1 (0.06)	0 (0)	0 (0)	−0.26
		**All**	**60**	**50** **(0.19)**	**1** **(0.2)**	**4** **(0.25)**	**0** **(0)**	**5** **(0.18)**	0.14
	Inconclusive	Autosomal dominant	11	7 (0.03)	1 (0.2)	1 (0.06)	1 (0.33)	1 (0.04)	1.22
		Autosomal recessive	6	5 (0.02)	0 (0)	1 (0.06)	0 (0)	0 (0)	−0.26
		X-linked	2	0 (0)	0 (0)	0 (0)	0 (0)	2 (0.07)	4.09*
		**All**	**19**	**12** **(0.05)**	**1** **(0.2)**	**2** **(0.13)**	**1** **(0.33)**	**3** **(0.11)**	2.04
	Negative	Negative	237	202 (0.77)	3 (0.6)	10 (0.63)	2 (0.67)	20 (0.71)	
	Total		316	264	5	16	3	28	
European	Positive	Autosomal dominant	39	6 (0.1)	5 (0.1)	6 (0.14)	8 (0.12)	14 (0.15)	0.75
		Autosomal recessive	15	5 (0.09)	2 (0.04)	0 0)	3 (0.04)	5 (0.05)	−0.58
		X-linked	6	1 (0.02)	0 (0)	1 (0.02)	1 (0.01)	3 (0.03)	0.88
		**All**	**60**	**12** **(0.21)**	**7** **(0.13)**	**7** **(0.16)**	**12** **(0.18)**	**22** **(0.23)**	0.56
	Inconclusive	Autosomal dominant	11	2 (0.03)	3 (0.06)	3 (0.07)	2 (0.03)	1 (0.01)	−1.26
		Autosomal recessive	6	1 (0.02)	2 (0.04)	1 (0.02)	0 (0)	2 (0.02)	−0.48
		X-linked	2	2 (0.03)	0 (0)	0 (0)	0 (0)	0 (0)	−2.18
		**All**	**19**	**5** **(0.09)**	**5** **(0.1)**	**4** **(0.09)**	**2** **(0.03)**	**3** **(0.03)**	−1.89
	Negative	Negative	237	41 (0.71)	40 (0.77)	33 (0.75)	54 (0.79)	69 (0.73)	
	Total		316	58	52	44	68	94	
Middle Eastern	Positive	Autosomal dominant	39	29 (0.12)	9 (0.15)	1 (0.2)	0 (0)	0 (0)	0.12
		Autosomal recessive	15	11 (0.04)	3 (0.05)	0 (0)	0 (0)	1 (0.25)	1.20
		X-linked	6	5 (0.02)	1 (0.02)	0 (0)	0 (0)	0 (0)	−0.44
		**All**	**60**	**45** **(0.18)**	**13** **(0.21)**	**1** **(0.2)**	**0** **(0)**	**1** **(0.25)**	0.54
	Inconclusive	Autosomal dominant	11	10 (0.04)	1 (0.02)	0 (0)	0 (0)	0 (0)	−1.02
		Autosomal recessive	6	5 (0.02)	0 (0)	0 (0)	0 (0)	1 (0.25)	1.55
		X-linked	2	2 (0.01)	0 (0)	0 (0)	0 (0)	0 (0)	−0.66
		**All**	**19**	**17** **(0.07)**	**1** **(0.02)**	**0** **(0)**	**0** **(0)**	**1** **(0.25)**	−0.05
	Negative	Negative	237	183 (0.75)	48 (0.77)	4 (0.8)	0 (0)	2 (0.5)	
	Total		316	245	62	5	0 (0)	4	
South Asian	Positive	Autosomal dominant	39	36 (0.12)	0 (0)	2 (0.4)	0 (0)	1 (0.06)	−0.32
		Autosomal recessive	15	13 (0.04)	0 (0)	0 (0)	0 (0)	2 (0.11)	1.05
		X-linked	6	6 (0.02)	0 (0)	0 (0)	0 (0)	0 (0)	−0.66
		**All**	**60**	**55** **(0.19)**	**0** **(0)**	**2** **(0.4)**	**0** **(0)**	**3** **(0.17)**	0.07
	Inconclusive	Autosomal dominant	11	10 (0.03)	0 (0)	0 (0)	0 (0)	1 (0.06)	0.36
		Autosomal recessive	6	5 (0.02)	0 (0)	1 (0.2)	0 (0)	0 (0)	0.19
		X-linked	2	2 (0.01)	0 (0)	0 (0)	0 (0)	0 (0)	−0.38
		**All**	**19**	**17** **(0.06)**	**0** **(0)**	**1** **(0.2)**	**0** **(0)**	**1** **(0.06)**	0.26
	Negative	Negative	237	220 (0.75)	1 (1)	2 (0.4)	0 (0)	14 (0.78)	
	Total		316	292	1	5	0 (0)	18	

*Indicates a C-A test Z-statistic with *P* value < 0.0007.

Note: Values in bold indicate the combined Autosomal dominant, Autosomal recessive, and X-linked cases.

The results of the logistic regression analyses largely mirrored the Cochran-Armitage test results (Supplementary Table 1). The coefficient of the indicator variable for prenatal vs pediatric ranged from −0.187 to −0.201 reflecting a diagnostic yield ratio of 0.62–0.65 for the prenatal versus pediatric cases. None of the beta coefficients for genetic ancestry was statistically significant. To gauge the power of the ancestry tests, we also calculated a 95% confidence interval for each ancestry beta from its mean and standard error. For each ancestry, using the model parameters, we calculated the probability of a positive outcome at 0% ancestry (P0) and then at 100% ancestry with the lower and upper 95% CI beta values (PL and PU, respectively). We then calculated the ratios Ratio-Lower = PL/P0 and Ratio-Upper = PU/P0 (final two columns of Supplementary Table 1). For each ancestry, the range of Ratio-Lower to Ratio-Upper is broad and includes 1. However, the range is broadest for African and Middle Eastern genetic ancestry. The ranges reflect the standard errors of beta, which are largest for African and Middle Eastern genetic ancestry, smallest for European and Native American genetic ancestry and intermediate for East and South Asian genetic ancestries. These standard errors (and hence power) are also a direct reflection of the observed variance in the genetic ancestries (mentioned above), which are largest for European and Native American, smallest for African and Middle Eastern, and intermediate for East and South Asian genetic ancestries.

### Genetic ancestry and diagnostic yield stratified by mode of inheritance and inheritance pattern

Similarly, there was no statistically significant reduction in positive cases compared to negative cases associated with any estimated genetic ancestry in pediatric or prenatal cases, when the positive cases were stratified by mode of inheritance (AD, AR, XL) (Table 2). In contrast, there was a significant association of East Asian genetic ancestry with XL inheritance among inconclusive prenatal cases. However, this was due entirely to 2 inconclusive cases of XL inheritance in the highest bin of East Asian ancestry, whereas no such association was observed in pediatric inconclusive cases. There was also a statistically significant association between estimated Middle Eastern ancestry and AR inheritance among pediatric inconclusive cases, and a similar trend in this direction in the prenatal inconclusive cases, although numbers were quite small.

We further broke down the AR cases into homozygotes and compound heterozygotes (Supplementary Table 2, Supplementary Table 3). The association of inconclusive pediatric AR outcomes with Middle Eastern ancestry was observed only among homozygous outcomes, and not compound heterozygotes (Supplementary Table 2). Among prenatal cases, we again saw a positive association of Middle Eastern ancestry with both positive and inconclusive homozygous AR outcomes. A similar pattern was observed with South Asian ancestry. In the pediatric cases, South Asian ancestry was positively associated with both positive and inconclusive homozygous AR outcomes (Supplementary Table 2) but only a modest positive trend in the prenatal cases (Supplementary Table 3).

### Consanguinity coefficient and diagnostic yield

Overall, 14.3% of the 845 total cases had an F > = 0.0156, the level for offspring of second cousins (Supplementary Fig. 4). Both positive and inconclusive AR (homozygous) cases were associated with an increased consanguinity coefficient (F) among the combined pediatric and prenatal cases (Table 3). There was a statistically significant increase in mean F in AR (homozygous) outcomes among both positive and inconclusive cases compared to negative cases by unpaired *t* test (*P* value < 0.0042).

**Table 3:** Mean and standard error (SE) of consanguinity coefficients (F) by Inheritance pattern in 845 P^3^EGS cases.

Outcome	Inheritance pattern	*N*	Mean F	SE	T-test statistic	*P* value
Positive	Autosomal dominant de novo	102	−0.0076	0.0024	−1.64	>0.1
	Autosomal dominant inherited	18	−0.00734	0.0031	−1.28	>0.1
	Autosomal dominant unknown	18	−0.00323	0.0048	−0.05	>0.1
	Autosomal recessive (compound heterozygous)	22	−0.0148	0.0090	−1.30	>0.1
	Autosomal recessive (homozygous)	18	0.043	0.0115	3.98	0.00092*
	X-linked	23	−0.00386	0.0037	−0.22	>0.1
Negative	Negative	552	−0.00298	0.0014		
Inconclusive	Autosomal dominant de novo	18	0.000677	0.0080	0.45	>0.1
	Autosomal dominant inherited	15	−0.0102	0.0068	−1.04	>0.1
	Autosomal dominant unknown	8	−0.00969	0.0058	−1.12	>0.1
	Autosomal recessive (compound heterozygous)	11	−0.0204	0.0071	−2.41	0.035
	Autosomal recessive (homozygous)	27	0.0494	0.0111	4.68	7.21E-05*
	X-linked	13	0.00925	0.0105	1.15	>0.1

*Indicates *P* value < 0.0042.

### Consanguinity coefficient and race/ethnicity

We examined the AR homozygous positive and inconclusive cases by self-reported race/ethnicity of the parents and consanguinity coefficient of the proband, as well as the variant frequencies. Among 18 positive cases, 10 had consanguinity coefficients greater than 0.0156 (average of 0.075, minimum 0.022, maximum 0.171). For 3 of these cases the parents were South Asian, in two cases the parents were Central Asian, in 2 cases the parents were Middle Eastern, in 2 cases the parents were Latino(a) and in one case the parents were East Asian race/ethnicity. Among 8 cases with consanguinity coefficients less than 0.0156 (average of 0.003), 5 had parents that were Latino(a), and one each were Central Asian, African American or Black, and white/European race/ethnicities. For all cases, the frequencies of P and LP variants estimated from gnomAD (based on the genetic ancestry estimates of the proband) were uniformly low; all were below 0.0001 except for a non-consanguineous Central Asian (0.00014) and non-consanguineous African American or Black (0.00041) case. It is notable that among the 10 cases with consanguinity, 7 were Middle Eastern, Central or South Asian, while among the 8 cases without consanguinity, 1 case was Middle Eastern, Central or South Asian race/ethnicities.

Among 27 AR homozygous inconclusive cases (with VUSs), 17 had consanguinity coefficients greater than .0156 (average of 0.081, minimum 0.023, maximum 0.21). In this group, for 8 cases the parents were Latino(a), in 5 cases the parents were Middle Eastern, in 2 cases each the parents were Central Asian and South Asian, and in one case the parents were East Asian race/ethnicity. In contrast, among 10 cases with consanguinity coefficients less than 0.0156 (average of −0.005), for 6 the parents were Latino(a), for 2 the parents were South Asian, and in one each the parents were white/European and African American or Black race/ethnicities. Here again it is notable that among the 17 cases with consanguinity, 9 had parents who identified as Middle Eastern, Central Asian, or South Asian, while among the 10 cases without consanguinity, the parents identified with these racial/ethnic groups for only 2. For these inconclusive AR homozygous cases, the P/LP allele frequencies were again all below 0.0001 in frequency except in 2 cases, one East Asian consanguineous case with allele frequency 0.0032, and one Latino(a) non-consanguineous case with allele frequency 0.00023. These variants represent ancestry-specific founder variants.

### Recurrent variants in the P^3^EGS cohort

In searching for possible founder variants, we found four recurrent variants in three different genes among eight different cases (Supplementary Table 4). Of note, the recurrent variants were all de novo, and therefore do not represent founder variants.

## Discussion

Among both pediatric and prenatal cases, we observed no reduction in overall diagnostic yield (definitive+ probable positive) from ES associated with any of the estimated genetic ancestry groups (African, Native American, East Asian, European, Middle Eastern, South Asian). Similarly, there was no reduction or increase in the rate of inconclusive outcomes associated with any of the genetic ancestries, with the single exception of a positive association with Middle Eastern genetic ancestry. Of 9 pediatric cases with primarily (> 87.5%) Middle Eastern genetic ancestry, 7 (78%) had an inconclusive result, including 2 AD, 4 AR and 1 XL, compared to 12% for the rest of the cohort. There were 4 prenatal cases with majority Middle Eastern genetic ancestry; 1 of these had an inconclusive result (AR).

The mode of inheritance distribution also differed significantly between positive and inconclusive outcomes, with a higher proportion of AD de novo results for positive versus inconclusive cases^7^, likely a direct reflection of the American College of Medical Genetic and Genomics (ACMG) criteria, for which de novo status of a variant is considered a primary criterion for pathogenicity determination. Most of our cases that were classified as inconclusive were due to variant uncertainty^7^, and the majority of these VUSs were inherited variants or inheritance uncertain. We also observed a shift in mode of inheritance by genetic ancestry among our cases. AR homozygous inheritance was positively associated with Middle Eastern and South Asian genetic ancestry among both positive and inconclusive pediatric and prenatal cases. We also showed that these trends were largely due to consanguinity associated with these ancestries. Thus, while the overall diagnostic yield was not diminished in any non-European genetic ancestry, the pattern of inheritance varied. And the sole positive association of the inconclusive rate with Middle East genetic ancestry was largely attributable to 5 AR homozygous cases.

Some studies have suggested that diagnostic yield from ES and other genetic tests is lower in non-white race/ethnicity groups, such as African American, or Native American^3,9^ possibly due to underrepresentation of data from non-white populations in genetic variant databases^2,3,10^. However, the clinical context is important in evaluating the association of race/ethnicity or genetic ancestry with diagnostic yield. For example, in genetic testing studies of hearing loss in which children underwent comprehensive genetic testing (CGT) and panel testing, Hispanic/Latino(a) and African American children were less likely to have a definitive genetic diagnosis compared to white or Asian children^5,11^. This was due to the fact that likely causal variants in the African American and Latino(a) children had not yet been documented in prior studies (and therefore also not appearing on variant-specific panels), in contrast to some of the more common causal variants found in white and Asian children. When the authors reduced the ACMG criterion of prior association with disease and solely used in silico functional prediction, there was no difference in diagnostic yield by ancestry.

It appears that the requirement for prior evidence regarding a specific variant (as opposed to predicted functional evidence) can have a significant impact on diagnostic yield; an example from newborn screening demonstrated a reduction of diagnostic yield from 88 to 55% when requiring prior curation of a variant as P/LP as opposed to functional prediction with no prior curation, yet without a dramatic effect on false positive rate (increase from 0.6 to 1.6%)^12^. It is therefore important to consider the role of prior evidence of pathogenicity or likely pathogenicity for a variant in assessing genetic ancestry influences on diagnostic yield, as lack of inclusion of some ancestral groups in clinical genetic studies may lead to underrepresentation of ancestry-specific pathogenic founder mutations in clinical variant databases.

In our study, cases were selected with a broad range of clinical phenotypes, with no prior assumptions about potential mode of inheritance. The majority of our positively diagnosed pediatric and prenatal cases were due to P/LP variants in AD genes (69%), and the majority of the variants were de novo (74% confirmed but possibly as high as 87% due to inheritance uncertainty). All of our XL cases were also dominant, and the majority arose de novo^7^. By contrast, 9% of the positive cases were due to inherited AD variants, and 20% had AR variants, nearly all of which were inherited. As expected, we observed no genetic ancestry association with de novo variants as these presumably occur independently of an individual’s genetic ancestry. However, we also saw no genetic ancestry associations in the inherited AD or AR cases. This was largely a reflection that nearly all variants were quite rare (frequency <0.0001), and, with few exceptions, likely did not reflect founder mutations in any of the conditions or groups studied. The possible exceptions are variants observed in AR homozygous cases with low consanguinity coefficients as well as AR compound heterozygotes. Of note, we found no genetic ancestry associations or even trends for the pediatric or prenatal AR compound heterozygous cases. On the other hand, we did observe an excess of Native American genetic ancestry among 9 AR homozygotes without consanguinity, reflecting that 6 of the 9 had parents who self-reported Latino(a) race/ethnicity, and suggesting the possibility of founder variants in some Native American indigenous populations.

Among the inconclusive cases, the proportion of inherited cases is substantially higher at 58% (53/92). Yet here also, we found no association with any of the genetic ancestries tested for the inherited AD and compound heterozygous AR cases. Again, this suggests that while variant uncertainty may have led to this collection of outcomes, there was no bias towards non-European ancestries, likely because of the lack of elevated frequency of founder variants underlying the disorders identified. In the entire cohort, we identified only one P/LP/VUS variant with increased frequency—the AR VUS c.636 G > C (p.[Gln212His]) (rs201590882) in *ARMC9* in an East Asian case (gnomAD frequency of 0.003 in East Asians). Furthermore, the four variants found twice among our cases were all de novo and not inherited.

The increased AR homozygous inheritance cases in high Middle Eastern and South Asian genetic ancestry pediatric cases, corresponding with statistically increased estimated consanguinity coefficients, was expected. It is well documented that certain population groups such as those from the Middle East, and South and Central Asia, have increased F, which increases autozygosity and hence the rate of AR homozygous cases^13,14^.

We also note that our diagnostic yield and results on ancestry are a direct reflection of the clinical setting of rare, undiagnosed diseases and implementation of the ACMG criteria for variant annotation, as well as our inclusion/exclusion criteria. Our inclusion criteria required a prior negative microarray, and all patients with a prior positive genetic test (e.g., from a gene-based panel) were also excluded. Thus, the diagnostic yield of 26.7% for our pediatric cases may be lower than other studies with different diagnostic and inclusion/exclusion criteria but comparable to others with similar criteria.

The ACMG criteria place a special emphasis on de novo inheritance, leading to a higher proportion of de novo AD variants in positive cases compared to inconclusive cases in our study. While there was a lack of founder variants underlying the genetic etiology of the cases in our study, this phenomenon may not be general—for example in the study of known predominantly AR diseases (such as hearing loss or inborn errors of metabolism), where ancestry associations may still be present depending on variant annotation requirements. Thus, our results should not necessarily be considered representative of all clinical testing scenarios. Indeed, our results contrast with other scenarios, such as gene panels and polygenic risk scores, which typically involve more common and genetic-ancestry-specific variants, where the impact of detection biases favoring European as opposed to other genetic ancestries has been well documented^5,15^.

In summary, in this ancestrally diverse cohort of pediatric and prenatal cases with different clinical indications, there was no reduction in diagnostic yield associated with any genetic ancestry group. Consanguinity may increase the relative proportion of cases with AR homozygous inheritance among those with Middle Eastern and South Asian genetic ancestry but did not alter the overall diagnostic yield, although our number of cases with these ancestries was modest. This empirical study improves our understanding and provides support for the equitable use of exome sequencing in diagnosis of previously undiagnosed but potentially Mendelian disorders across all ancestral populations.

## Methods

### Study participants, recruitment, demographics, inclusion, and exclusion criteria

Pediatric (*N* = 529) and prenatal (*N* = 316) cases and their available biological parents (at least one required) were primarily enrolled at one of five sites in the San Francisco Bay area and Central Valley of California. The five sites included UCSF Benioff Children’s Hospital San Francisco and Benioff Children’s Hospital Oakland, Zuckerberg San Francisco General Hospital, the Betty Irene Moore Women’s Hospital at Mission Bay, and the Community Medical Center in Fresno.

The study was approved by the UCSF Institutional Review Board (IRB) (protocols 17-22504 and 17-22420), the Fresno Community Medical Center IRB (protocol 2019024), and was registered as two clinical trials (“Clinical Utility of Pediatric Whole Exome Sequencing”, NCT03525431 and “Clinical Utility of Prenatal Whole Exome Sequencing”, NCT03482141). Written informed consent was provided by adult participants >18 years of age, or by parents or legal guardians on behalf of their children <18 years of age or >18 years of age who were unable to consent independently. Assent was obtained from minors and intellectually disabled adults whenever possible. This study complied with all relevant ethical regulations including the Declaration of Helsinki. The study period was from 8/1/2017 to 5/13/2022.

ES was offered to patients seen in clinic for whom a genetic etiology was suspected based on clinical findings. A minimum of one biological parent was required to be available and willing to provide a biospecimen for ES, with a preference for two available parents. It was required that least one parent consented to ES of the child. For the prenatal cases, at least the mother had to consent to ES of a fetal sample as well as on herself. Pediatric patients were enrolled with the following indications: multiple congenital anomalies (MCAs), developmental delays (DD)/ intellectual disability (ID), metabolic disease, epilepsy, seizures, neurodegenerative disease/cerebral palsy (CP), and encephalopathy. Pediatric patients must have had at least one prior genetics appointment or evaluation. Almost all pediatric patients were resident in California and were likely to have had non-diagnostic newborn screening prior to enrollment. Specific community outreach efforts for patient recruitment were not required for the pediatric patients, as the patient population seen at the Benioff Children’s Hospitals in San Francisco and Oakland was diverse.

Pregnant women with fetuses with structural birth defects identified by ultrasound were enrolled. The prenatal eligibility criteria included: one or more fetal structural abnormalities, an unexplained disorder of fetal growth, and one or more fetal effusions or non-immune hydrops. This was based on imaging at the time of enrollment. All prenatal cases had to have undergone prenatal diagnosis with non-diagnostic chromosomal microarray. Pregnant patients late in gestation, in whom ES results were not anticipated until after delivery, were included in the prenatal subgroup if consent occurred prior to delivery. Twin gestations were eligible for inclusion if one or both fetuses were affected.

As an exclusion criterion, patients with a diagnosis that explained their clinical findings after microarray were excluded from the study. Microarrays were ordered for patients with multiple anomalies, DD/ID, and/or autism prior to study entry. Microarrays were also ordered for growth delays, including short stature, failure to thrive or microcephaly, and neurological findings such as hypotonia and seizures. A modification of the guidelines of Manning et al.^16^ was used for the microarrays ordered. Pregnancies and patients with a copy number variant not clearly associated with the phenotype were eligible for inclusion, as were patients who had previously undergone targeted or gene panel testing without a diagnosis. Patients were excluded from the study if both biological parents were unavailable or if prior ES was performed for a clinical or research indication. Patient recruitment, inclusion and exclusion has also been described in ref. ^7^.

### Self-reported race/ethnicity/nationality of parents

Parents of affected probands voluntarily responded to questions about their demographic background on a structured instrument. In terms of race/ethnicity/nationality, the P^3^EGS parents were asked to respond to all categories that best describe them among: (a) American Indian, Native American or Alaska Native, (b) Asian-Filipino, (c) Asian-Central/South Asian (Indian, Pakistani, Afghani), (d) Asian-Vietnamese, (e) Asian-Hmong, (f) Asian-Korean, (g) Asian-Japanese, (h) Asian-other (specified through free text), (i) Black or African American, (j) Native Hawaiian, (k) Samoan, (l) Other Pacific Islander (specified through free text), (m) white or European American, (n) Middle Eastern or North African/Mediterranean, (o) Hispanic/Latino(a) – Mexican, Mexican American, Chicano/a, (p) Hispanic/Latino(a) – Central American -Guatemala, El Salvador, etc., (q) Hispanic/Latino(a) – South American -Peru, Chile, etc., (r) Hispanic/Latino(a) – Caribbean -Puerto Rico, Cuba, etc., (s) Hispanic/Latino(a) – another Hispanic or Latino origin (specified by free text), (t) Prefer not to answer (u) Unknown/none of these fully describe them. They also responded to the open-ended questions “What is your ancestry or ethnic origin?” and “What country were you born in?”

Based on the parental responses to the demographic questionnaires, we derived the following categories (based primarily on the selected pre-listed categories above, and further resolved using the open-ended questions): Native American (NAT) —based on category (a); Latino(a) (LT)—based on categories (o–s) which were rolled up; white/European (EU)—based on category m); African American or Black (AF)—based on category (i); East Asian (EA)—based on categories (b, d–g) which were rolled up; South Asian (SA) and Central Asian (CA)—by separating category (c) into SA and CA based on information from the open-ended questions on ancestry and country of origin; Middle Eastern (ME)—based on category (n) and Pacific Islander (PI)—based on categories (j–l) which were rolled up. The open-ended questions were also used to resolve category (h) into EA, SA, or CA. Each of the parents was placed in one or more of the categories or “missing” if no information was provided.

We only included self-reported race/ethnicity categories for parents, as no self-report information is available for children or fetuses, and parents did not assign race/ethnicity categories to their offspring.

### Exome sequencing, quality control and selection of markers for genetic ancestry analyses

ES of samples from the probands and available parents was done at UCSF, in a Clinical Laboratory Improvement Amendments (CLIA) licensed laboratory, the UCSF Clinical Cancer Genomics Laboratory (CLIA number: 05D2034158). Written, informed consent was obtained for study participation. Initially, ES was provided to probands and both biological parents if both parents were available. Duo ES was provided in cases where only one biological parent was available. However, in the last year of enrollment, a ‘proband first’ approach was used, and biological parents only underwent targeted Sanger sequencing if segregation analysis was required^7^.

Exon regions were targeted using the xGen Whole Exome Panel kit from Integrated DNA Technologies. The targeted regions were sequenced using the Illumina HiSeq 2500 sequencing system (v3 chemistry) with 100 bp paired end reads in rapid run mode. The DNA sequences were aligned to the reference published human genome GRch37 (see ref. ^7^ for full methods).

Variants in all VCF files with sequencing depth at or below 10 (DP < = 10), and genotype quality equal to or less than 20 (GQ < = 20) were filtered out using GATK^17^. The VCF files were then lifted over from human genome reference version GRCh37 to GRCh38 using the Picard tool in GATK suite of tools^17^. Human Genome Diversity Panel (HGDP) whole genome sequencing samples from the GnomAD V3 call set^18,19^ were used as the reference for genetic ancestry and admixture estimation (*N* = 829 unrelated individuals). The HGDP samples were all mapped to the GRCh38 reference sequence.

High-performance markers were selected from the HGDP and P^3^EGS data for downstream genetic ancestry, admixture, relatedness, and consanguinity analysis using the following criteria:

1) Restriction of markers in the HGDP dataset to exome sequenced regions in the P^3^EGS dataset. This was conducted using bcftools^20^.

2) MAF > = 0.05 in any of 7 supergroups in GnomAD HGDP unrelated individuals:

(i) African, (ii) Native American, (iii) South Asian, (iv) East Asian, (v) European, (vi) Middle Eastern, (vii) Oceanian.

3) Only biallelic, autosomal SNPs, with a call rate >95% in exome regions that were sequenced were selected (This was done in both HGDP and P^3^EGS cohorts separately). The resulting markers in the HGDP cohort (*N* = 105,956) were intersected with markers from the P^3^EGS cohort sample VCFs, resulting in *N* = 95,173 markers. Variants in regions known to affect principal components (PCs) (HLA region on chromosome 6p, inversion on chromosome 8p23 and inversion on chr 17q21, GRCh38 build) were removed resulting in 82,349 markers after filtering. After linkage disequilibrium pruning (0.5 kb in a 5000 kb window), 53,665 high-performance markers for principal components analysis and genetic admixture analysis remained.

### Genetic ancestry, admixture, relatedness, and consanguinity analysis

Individual genetic ancestry admixture proportions were estimated using the ADMIXTURE software package^21^ using the set of 53,665 exome-wide markers. A supervised approach, whereby unrelated individuals in (K) reference populations are assumed to have 100% reference genetic ancestry, was utilized to estimate the individual genetic admixture proportions in individuals of the P^3^EGS cohort (parents and probands). We created K = 7 reference continental/subcontinental ancestral populations from the Human Genome Diversity Panel (HGDP) individuals, based on literature^22^. The 7 reference populations were: African–Afr (Yoruba, Mandenka *N* = 40), Native American–Amr (Colombia, Karitiana, Surui, Pima, *N* = 40); East Asian–Eas (Han, Japanese *N* = 40); Middle Eastern–Mid (Druze, Palestinian, Bedouin, *N* = 40); European-Eur (French, Orcadian, Tuscan, Sardinian, *N* = 40); Oceanian–Oce (Papuan, Melanesian *N* = 30); South Asian–Sas (Pathan, Sindhi, *N* = 39). The genetic ancestry admixture proportions were visualized using Pong^23^.

Principal components analysis (PCA) was also performed on the HGDP samples using the SmartPca program, part of the EIGENSOFT4.2 software package^24^ using the same 53,665 markers. The P^3^EGS samples were then projected onto the HGDP-derived PCs to facilitate geographic interpretation of the P^3^EGS participants PCs.

Genetic kinship between P^3^EGS participants (probands, parents) was estimated using PC-Relate^25^. Genetic ancestry was controlled by using the first 8 PCs from PCA in PC-Relate in order to estimate only recent genetic relatedness due to family structure. Linkage disequilibrium (LD) pruning (0.1 kb in a 1000 kb window) was done to select a set of independent SNPs for the relatedness analysis including the PCA used for control of ancestry. Similarly, consanguinity coefficients for probands were estimated using PC-relate, also controlling for ancestry using the first eight PCs. Consanguinity coefficient (F) is the probability that two alleles at a locus in an individual are identical by descent from a common ancestor. Children of 1st cousins have a consanguinity coefficient of 1/16 (0.0625), while of 2nd cousins it is 1/64 (0.0156).

### Clinical/diagnostic outcomes or case classification

Cases received one of either a positive (definitive positive, probable positive), inconclusive or negative case outcome based on identification of pathogenic (P), likely pathogenic (LP), variant of uncertain significance (VUS) or no primary variant(s) found (see ref. ^7^ for more details).

### Statistical analyses

The primary analysis was to compare the distribution of genetic ancestries in positive, inconclusive, and negative cases. A Kolmogorov–Smirnov test was used to examine the difference in empirical cumulative distribution functions (ECDFs) of estimated genetic ancestries in positive, inconclusive, and negative cases separately for the pediatric and prenatal cases. The tests were conducted over all modes of inheritance, then stratified by mode of inheritance—Autosomal Dominant (AD), Autosomal Recessive (AR), X-linked (XL), and finally stratified by inheritance pattern (AD de novo, AD inherited, AD inheritance unknown, AR homozygous, AR compound heterozygous, XL). The significance thresholds were *P* value < 0.002, *P* value < 0.0007, *P* value < 0.0003 for the overall, mode of inheritance and inheritance pattern stratified analyses to account for multiple testing (Bonferroni correction). The Bonferroni significance thresholds were obtained by dividing the original alpha level/threshold of 0.05 by the number of tests performed. For the overall analyses comparing positive vs negative, and inconclusive vs negative across 6 ancestries, 24 tests were conducted. For the mode of inheritance analyses, 72 tests were conducted, and finally in the inheritance pattern analysis, 144 tests were conducted.

Because of non-normality and discontinuity in the genetic ancestry distributions, we created five bins of genetic ancestry as follows: 0–12.5%; >12.5–37.5%; >37.5–62.5%; >62.5–87.5%; >87.5–100%. These intervals were selected to reflect ranges of values around number of grandparents of differing genetic ancestry (i.e., 0, 1, 2, 3, 4). Non-parametric Cochran-Armitage trend tests were also conducted to determine whether there was a linear trend between diagnostic yield and estimated genetic ancestry (in the genetic ancestry bins). The negative cases were used as the control/reference. This was repeated in analyses stratified by mode of inheritance and inheritance pattern. To account for multiple testing, the significance thresholds were *P* value < 0.002, *P* value < 0.0007, *P* value < 0.0003 for the overall, mode of inheritance and inheritance pattern stratified analyses, similar to the correction done in the Kolmogorov–Smirnov tests above. All tests conducted above were two-tailed.

As a complement to the above tests of association and to combine the pediatric and prenatal results, we also performed logistic regressions of diagnostic yield (1 for positive, 0 for all other) for each genetic ancestry as an independent predictor variable. Regressions were conducted for pediatric and prenatal cases together by including an indicator variable for prenatal/pediatric as an additional independent covariate.

Unpaired *t* tests were used to compare means of estimated consanguinity coefficients between positive/ inconclusive cases stratified by inheritance pattern vs negative cases (Bonferroni corrected significance threshold *P* value < 0.0042, where 12 two-tailed *t* tests were performed with an original alpha level of 0.05).

### Reporting summary

Further information on research design is available in the Nature Research Reporting Summary linked to this article.

### Supplementary information

REPORTING SUMMARY

Supplementary tables and figures

## Data Availability

Data has been uploaded to the database of Genotypes and Phenotypes (dbGaP); study Accession: phs002324.v3.p1. Clinical data is available from the authors on reasonable request. The sequencing was performed in a Clinical Laboratory Improvement Amendments (CLIA) licensed laboratory, the UCSF Clinical Cancer Genomics Laboratory (CLIA number is: 05D2034158).

## Code availability

The underlying code for this study is not publicly available but may be made available to qualified researchers on reasonable request from the corresponding author. R software, version 4.1.2 was used for the statistical calculations.
