# Supplementary material for: Genetic ancestry and diagnostic yield of exome sequencing in a diverse population
Source: NPJ Genom Med. 2024 Jan 3;9:1. doi: 10.1038/s41525-023-00385-6 (PMC10764913; doi:10.1038/s41525-023-00385-6)
Supplement: Supplementary file 1 — REPORTING SUMMARY [file 41525_2023_385_MOESM1_ESM.pdf]

nature portfolio

Corresponding author(s): Yusuph Mavura, Neil Risch

Last updated by author(s): Nov 2, 2023

Reporting Summary

Nature Portfolio wishes to improve the reproducibility of the work that we publish. This form provides structure for consistency and transparency in reporting. For further information on Nature Portfolio policies, see our Editorial Policies and the Editorial Policy Checklist.

Statistics

For all statistical analyses, confirm that the following items are present in the figure legend, table legend, main text, or Methods section.

n/a	Confirmed	
☐	☒	The exact sample size (*n*) for each experimental group/condition, given as a discrete number and unit of measurement
☐	☒	A statement on whether measurements were taken from distinct samples or whether the same sample was measured repeatedly
☐	☒	The statistical test(s) used AND whether they are one- or two-sided<br>*Only common tests should be described solely by name; describe more complex techniques in the Methods section.*
☐	☒	A description of all covariates tested
☐	☒	A description of any assumptions or corrections, such as tests of normality and adjustment for multiple comparisons
☐	☒	A full description of the statistical parameters including central tendency (e.g. means) or other basic estimates (e.g. regression coefficient) AND variation (e.g. standard deviation) or associated estimates of uncertainty (e.g. confidence intervals)
☐	☒	For null hypothesis testing, the test statistic (e.g. *F*, *t*, *r*) with confidence intervals, effect sizes, degrees of freedom and *P* value noted<br>*Give P values as exact values whenever suitable.*
☒	☐	For Bayesian analysis, information on the choice of priors and Markov chain Monte Carlo settings
☒	☐	For hierarchical and complex designs, identification of the appropriate level for tests and full reporting of outcomes
☒	☐	Estimates of effect sizes (e.g. Cohen's *d*, Pearson's *r*), indicating how they were calculated

Our web collection on statistics for biologists contains articles on many of the points above.

Software and code

Policy information about availability of computer code

Data collection	Provided in text;Code for this study will be made available to qualified researchers on reasonable request from the corresponding authors.
Data analysis	Provided in text

For manuscripts utilizing custom algorithms or software that are central to the research but not yet described in published literature, software must be made available to editors and reviewers. We strongly encourage code deposition in a community repository (e.g. GitHub). See the Nature Portfolio guidelines for submitting code & software for further information.

Data

Policy information about availability of data

All manuscripts must include a data availability statement. This statement should provide the following information, where applicable:

- Accession codes, unique identifiers, or web links for publicly available datasets
- A description of any restrictions on data availability
- For clinical datasets or third party data, please ensure that the statement adheres to our policy

Data has been uploaded to the database of Genotypes and Phenotypes (dbGaP); study Accession: phs002324.v3.p1. Clinical data is available from the authors on reasonable request. The sequencing was performed in a Clinical Laboratory Improvement Amendments (CLIA) licensed laboratory, the UCSF Clinical Cancer Genomics Laboratory (CLIA number is: 05D2034158).

Research involving human participants, their data, or biological material

Policy information about studies with human participants or human data. See also policy information about sex, gender (identity/presentation), and sexual orientation and race, ethnicity and racism.

Reporting on sex and gender	Patients of all sexes and gender were included
Reporting on race, ethnicity, or other socially relevant groupings	In terms of race/ethnicity/nationality, parents were asked to respond to all categories that best describe them among: a) American Indian, Native American or Alaska Native, b) Asian-Filipino, c) Asian-Central/South Asian (Indian, Pakistani, Afghani), d) Asian-Vietnamese, e) Asian-Hmong, f) Asian-Korean, g) Asian-Japanese, h) Asian-other (specified through free text), i) Black or African American, j) Native Hawaiian, k) Samoan, l) Other Pacific Islander (specified through free text), m) white or European American, n) Middle Eastern or North African/Mediterranean, o) Hispanic/Latino(a) – Mexican, Mexican American, Chicano/a, p) Hispanic/Latino(a) – Central American -Guatemala, El Salvador, etc., q) Hispanic/Latino(a) – South American -Peru, Chile, etc., r) Hispanic/Latino(a) – Caribbean -Puerto Rico, Cuba, etc., s) Hispanic/Latino(a) – another Hispanic or Latino origin (specified by free text), t) Prefer not to answer u) Unknown/none of these fully describe them. They also responded to the open-ended questions “What is your ancestry or ethnic origin?” and “What country were you born in?”<br>Based on the parental responses to the demographic questionnaires, we derived the following categories (based primarily on the selected pre-listed categories above, and further resolved using the open-ended questions): Native American (NAT) — based on category a); Latino(a) (LT) — based on categories o) to s) which were rolled up; White-European (EU) — based on category m); African American or Black (AF) — based on category i); East Asian (EA) — based on categories b), d) to g) which were rolled up; South Asian (SA) and Central Asian (CA) — by separating category c) into SA and CA based on information from the open-ended questions on ancestry and country of origin; Middle Eastern (ME) — based on category n) and Pacific Islander (PI) — based on categories j) to l) which were rolled up. The open-ended questions were also used to resolve category h) into EA, SA, or CA. Each of the parents was placed in one or more of the categories or “missing” if no information was provided.<br><br>We only included self-reported race/ethnicity categories for parents, as no self-report information is available for children or fetuses, and parents did not assign race/ethnicity categories to their offspring.
Population characteristics	Patients were enrolled at the University of California, San Francisco (UCSF) Benioff Children’s Hospital Mission Bay and the Betty Irene Moore Women’s Hospital. Pediatric patients were also enrolled at the Zuckerberg San Francisco General Hospital, UCSF Benioff Children’s Hospital Oakland and the Community Medical Center in Fresno from August 2017 through April 2021. Prenatal patients were also recruited from collaborating groups across the country. Parental race and ethnicity information was obtained by self-report on a harmonized survey. Exome Sequencing (ES) was provided to pediatric and prenatal cases for whom a genetic etiology was suspected based on clinical findings, and/or prior genetic testing with microarray, single-gene or gene panel sequencing had failed to yield a diagnosis. ES of samples from the probands and available parents was done at the UCSF Genomic Medicine Laboratory (GML). Details of the sequencing, quality control and selection of markers for genetic ancestry analyses are provided in Supplementary Methods. Initially, ES was provided to probands and both biological parents if both parents were available. Duo ES was provided in cases where only one biological parent was available. However, in the last year of enrollment, a ‘proband first’ approach was used, and biological parents only underwent targeted Sanger sequencing if segregation analysis was required.<br>URM pediatric and prenatal cases were defined as having at<br>least one biological parent who self-identified as belonging to any non-white racial or ethnic minority group. If the information on one parent was missing, the child was considered URM if the responding parent was URM; if the responding parent was white or if information was missing for both parents, the self-identified race/ethnicity was considered unknown. Patients were defined as US if they fulfilled one or more of the following three criteria: (1) covered by MediCal health insurance (California’s Medicaid option for low-income families), (2) living in a medically underserved area (MUA), as determined by the home zip code collected from the electronic medical record belonging to the patient and according to the Health Resources and Services Administration (HRSA) shortage designation criteria as listed on their website, and (3) living in a health professional shortage area (HPSA), as determined by the home zip code collected from the electronic medical record belonging to the patient, according to the HRSA shortage designation criteria.
Recruitment	We offered testing to patients seen in clinic for whom exome sequencing was clinically indicated, with a priority for US and URM families. Pediatric patients were enrolled with the following indications: Multiple congenital anomalies (MCAs), developmental disability (DD)/intellectual disability (ID), metabolic disease, epilepsy, neurodegenerative disease/cerebral palsy (CP), and encephalopathy. Patients with MCA, metabolic disease, epilepsy, and neurodegenerative disease/CP were further categorized as having, or not having, ID. Prenatal eligibility criteria were based on imaging at the time of enrollment, and included one or more fetal structural abnormalities, an unexplained disorder of fetal growth, and one or more fetal effusions or non-immune hydrops. We supported the families with interpreting services and study staff who spoke Spanish. For the pediatric patients, the patient population seen at the Benioff Children’s Hospitals in San Francisco and Oakland was diverse and we did not require specific community outreach efforts for patient recruitment.<br>Inclusion criteria for the study was:1. Presenting clinical features suggestive of a genetic etiology, including intellectual disability, seizures, multiple congenital anomalies, metabolic conditions, and neurodegenerative conditions or idiopathic cerebral palsy; up to 80 of these patients will have encephalopathy or multiple congenital anomalies so that they may benefit from rapid exome sequencing in the Pediatric Intensive Care Unit or Neonatal Intensive Care Unit.<br>2. Pregnant women with fetuses with structural birth defects identified by ultrasound.<br>3. A minimum of one biological parent is available and willing to provide a biospecimen for ES, with a preference for two available parents. At least one parent consenting to ES of the child. For the prenatal cases, at least the mother has to consent to ES of a fetal sample as well as on herself.<br>4. Pediatric patients must have had at least one prior genetics appointment or evaluation.<br>5. All pediatric patients with a clinical indication for chromosomal microarray analysis (CMA) and all prenatal patients were required to have non-diagnostic CMA results prior to enrollment. Pregnancies and patients with a copy number variant not

clearly associated with the phenotype were eligible for inclusion, as were patients who had previously undergone targeted or gene panel testing without a diagnosis.
6. Pregnant patients late in gestation, in whom ES results were not anticipated until after delivery, were included in the prenatal subgroup if consent occurred prior to delivery.
7. Twin gestations were eligible for inclusion if one or both fetuses were affected.
Exclusion criteria for the study was:
1. Prior ES performed for a clinical or research indication
2. Lack of phenotypic indication of a likely underlying genetic etiology
3. Both biological parents are unavailable

Ethics oversight

The study was approved by the UCSF Institutional Review Board (IRB) (protocols 17-22504 and 17-22420), the Fresno Community Medical Center IRB (protocol 2019024), and was registered as two clinical trials ("Clinical Utility of Pediatric Whole Exome Sequencing", NCT03525431 and "Clinical Utility of Prenatal Whole Exome Sequencing", NCT03482141). Written informed consent was provided by adult participants >18 years of age, or by parents or legal guardians on behalf of their children <18 years of age or >18 years of age who were unable to consent independently. Assent was obtained from minors and intellectually disabled adults whenever possible.
The study was approved by the UCSF Institutional Review Board (IRB) (protocols 17-22504 and 17-22420), the Fresno Community Medical Center IRB (protocol 2019024), and was registered as two clinical trials ("Clinical Utility of Pediatric Whole Exome Sequencing", NCT03525431 and "Clinical Utility of Prenatal Whole Exome Sequencing", NCT03482141). Written informed consent was provided by adult participants >18 years of age, or by parents or legal guardians on behalf of their children <18 years of age or >18 years of age who were unable to consent independently. Assent was obtained from minors and intellectually disabled adults whenever possible.

Note that full information on the approval of the study protocol must also be provided in the manuscript.

Field-specific reporting

Please select the one below that is the best fit for your research. If you are not sure, read the appropriate sections before making your selection.

☒ Life sciences ☐ Behavioural & social sciences ☐ Ecological, evolutionary & environmental sciences

For a reference copy of the document with all sections, see nature.com/documents/nr-reporting-summary-flat.pdf

Life sciences study design

All studies must disclose on these points even when the disclosure is negative.

Sample size	Sample size was determined by the number of patients enrolled during the course of the study.
Data exclusions	There were no data exclusions in calculating the diagnostic yield.
Replication	We performed Sanger sequencing to verify variants identified on exome sequencing
Randomization	Subjects were not randomized.
Blinding	Investigators were not blinded.

Reporting for specific materials, systems and methods

We require information from authors about some types of materials, experimental systems and methods used in many studies. Here, indicate whether each material, system or method listed is relevant to your study. If you are not sure if a list item applies to your research, read the appropriate section before selecting a response.

Materials & experimental systems

n/a	Involved in the study	
☒	☐	Antibodies
☒	☐	Eukaryotic cell lines
☒	☐	Palaeontology and archaeology
☒	☐	Animals and other organisms
☐	☒	Clinical data
☒	☐	Dual use research of concern
☒	☐	Plants

Methods

n/a	Involved in the study	
☒	☐	ChIP-seq
☒	☐	Flow cytometry
☒	☐	MRI-based neuroimaging

Clinical data

Policy information about clinical studies

All manuscripts should comply with the ICMJE guidelines for publication of clinical research and a completed CONSORT checklist must be included with all submissions.

Clinical trial registration	The study was registered as two clinical trials (“Clinical Utility of Pediatric Whole Exome Sequencing”, NCT03525431 and “Clinical Utility of Prenatal Whole Exome Sequencing”, NCT03482141). The study was started on 8.1.2017 and completed on 5.13.2022.
Study protocol	Obtainable through UCSF IRB
Data collection	The study was started on 8.1.2017 and completed on 5.13.2022.
Outcomes	Outcome was the diagnostic yield of exome sequencing.
