# Supplementary material for: Genetic ancestry and diagnostic yield of exome sequencing in a diverse population
Source: NPJ Genom Med. 2024 Jan 3;9:1. doi: 10.1038/s41525-023-00385-6 (PMC10764913; doi:10.1038/s41525-023-00385-6)
Supplement: Supplementary file 2 — Supplementary tables and figures [file 41525_2023_385_MOESM2_ESM.pdf]

Supplementary Table 1. Results of logistic regression analysis of diagnostic yield by genetic ancestry. Regression coefficient for prenatal/pediatric indicator has pediatric as reference. Beta is the regression coefficient for genetic ancestry, coded in 1% increments. P-value is for test of statistical significance of Beta. Ratio refers to ratio of projected probability of positive case outcome for 100% vs 0% of the listed ancestry based on regression model; Ratio-Lower is based on the lower 95% confidence limit for Beta and Ratio-Upper is based on the upper 95% confidence limit for Beta.

Genetic Ancestry	Intercept	Prenatal/ Pediatric	Beta	SE (Beta)	P-value	Ratio-Lower	Ratio-Upper
African	-0.967	-.201	-.00667	.00552	.227	.224	1.330
Native American	-0.977	-.201	-.00121	.00306	.692	.564	1.386
East Asian	-1.007	-.187	-.00080	.00330	.809	.561	1.463
European	-1.130	-.208	.00280	.00247	.258	.858	1.640
Middle Eastern	-0.970	-.194	-.00505	.00609	.407	.234	1.572
South Asian	-1.026	-.194	.00216	.00368	.556	.676	1.801

Supplementary Table 2: Number of pediatric cases by genetic ancestry bins, stratified by extended inheritance pattern.

				Number of pediatric cases & (%) in each bin					
**Ancestry**	**Case definition**	**Inheritance Pattern**	**N**	**0-12.5%**	**12.5-37.5%**	**37.5-62.5%**	**62.5-87.5%**	**87.5-100%**	**C-A test Z-statistic**
**African**	Positive	Autosomal dominant de novo	68	62	2	1	2	1	-0.25
		Autosomal dominant inherited	14	12	1	0	1	0	0.25
		Autosomal dominant unknown	17	13	2	1	1	0	0.93
		Autosomal recessive (compound heterozygous)	11	9	1	1	0	0	0.17
		Autosomal recessive (homozygous)	14	13	1	0	0	0	-0.77
		X-linked	17	17	0	0	0	0	-1.23
	Inconclusive	Autosomal dominant de novo	12	11	1	0	0	0	-0.66
		Autosomal dominant inherited	11	8	2	1	0	0	0.56
		Autosomal dominant unknown	7	6	0	1	0	0	0.18
		Autosomal recessive (compound heterozygous)	8	7	0	0	0	1	0.94
		Autosomal recessive (homozygous)	24	23	0	0	1	0	-0.66
		X-linked	11	10	0	1	0	0	-0.21
	Negative	Negative	315	284	10	4	13	4	
**Native American**	Positive	Autosomal dominant de novo	68	28	9	21	6	4	0.26
		Autosomal dominant inherited	14	8	2	3	1	0	-1.25
		Autosomal dominant unknown	17	6	4	5	1	1	0.09
		Autosomal recessive (compound heterozygous)	11	6	2	3	0	0	-1.27
		Autosomal recessive (homozygous)	14	9	0	4	0	1	-1.03
		X-linked	17	7	1	8	1	0	-0.10
	Inconclusive	Autosomal dominant de novo	12	7	1	2	2	0	-0.80
		Autosomal dominant inherited	11	5	1	3	2	0	-0.06
		Autosomal dominant unknown	7	4	2	1	0	0	-1.35
		Autosomal recessive (compound heterozygous)	8	4	1	3	0	0	-0.75
		Autosomal recessive (homozygous)	24	12	1	7	2	2	0.01

		X-linked	11	5	0	5	1	0	-0.06
	Negative	Negative	315	136	43	85	37	14	
**East Asian**	Positive	Autosomal dominant de novo	68	61	2	5	0	0	-0.96
		Autosomal dominant inherited	14	14	0	0	0	0	-1.14
		Autosomal dominant unknown	17	14	1	1	0	1	0.50
		Autosomal recessive (compound heterozygous)	11	9	1	0	0	1	0.55
		Autosomal recessive (homozygous)	14	13	0	0	0	1	-0.02
		X-linked	17	14	1	0	0	2	0.97
	Inconclusive	Autosomal dominant de novo	12	12	0	0	0	0	-1.05
		Autosomal dominant inherited	11	9	0	1	0	1	0.85
		Autosomal dominant unknown	7	7	0	0	0	0	-0.81
		Autosomal recessive (compound heterozygous)	8	7	0	0	0	1	0.60
		Autosomal recessive (homozygous)	24	23	0	0	0	1	-0.62
		X-linked	11	11	0	0	0	0	-1.01
	Negative	Negative	315	283	8	4	4	16	
**European**	Positive	Autosomal dominant de novo	68	9	28	15	5	11	0.24
		Autosomal dominant inherited	14	0	5	3	2	4	1.88
		Autosomal dominant unknown	17	2	6	6	1	2	0.08
		Autosomal recessive (compound heterozygous)	11	1	4	2	0	4	1.24
		Autosomal recessive (homozygous)	14	6	6	1	0	1	-2.28
		X-linked	17	2	9	0	2	4	0.44
	Inconclusive	Autosomal dominant de novo	12	2	4	1	3	2	0.61
		Autosomal dominant inherited	11	3	4	2	1	1	-0.78
		Autosomal dominant unknown	7	0	1	3	1	2	1.77
		Autosomal recessive (compound heterozygous)	8	2	3	1	1	1	-0.38
		Autosomal recessive (homozygous)	24	10	12	0	1	1	-3.19
		X-linked	11	2	3	2	1	3	0.79
	Negative	Negative	315	58	115	63	28	51	
**Middle Eastern**	Positive	Autosomal dominant de novo	68	52	16	0	0	0	-0.28
		Autosomal dominant inherited	14	11	3	0	0	0	-0.28

		Autosomal dominant unknown	17	14	3	0	0	0	-0.60
		Autosomal recessive (compound heterozygous)	11	8	3	0	0	0	0.12
		Autosomal recessive (homozygous)	14	11	2	0	0	1	1.15
		X-linked	17	12	5	0	0	0	0.31
	Inconclusive	Autosomal dominant de novo	12	7	3	0	0	2	3.79
		Autosomal dominant inherited	11	9	1	0	1	0	0.66
		Autosomal dominant unknown	7	5	1	1	0	0	0.87
		Autosomal recessive (compound heterozygous)	8	5	3	0	0	0	0.65
		Autosomal recessive (homozygous)	24	15	5	0	0	4	4.49
		X-linked	11	8	2	0	0	1	1.70
	Negative	Negative	315	243	68	1	2	1	
**South Asian**	Positive	Autosomal dominant de novo	68	61	1	0	3	3	1.29
		Autosomal dominant inherited	14	14	0	0	0	0	-0.91
		Autosomal dominant unknown	17	17	0	0	0	0	-1.00
		Autosomal recessive (compound heterozygous)	11	11	0	0	0	0	-0.81
		Autosomal recessive (homozygous)	14	9	0	2	1	2	3.95
		X-linked	17	17	0	0	0	0	-1.00
	Inconclusive	Autosomal dominant de novo	12	12	0	0	0	0	-0.84
		Autosomal dominant inherited	11	11	0	0	0	0	-0.81
		Autosomal dominant unknown	7	7	0	0	0	0	-0.65
		Autosomal recessive (compound heterozygous)	8	8	0	0	0	0	-0.69
		Autosomal recessive (homozygous)	24	19	0	0	3	2	2.99
		X-linked	11	11	0	0	0	0	-0.81
	Negative	Negative	315	296	1	3	9	6	

Supplementary Table 3: Number of prenatal cases by genetic ancestry bins, stratified by extended inheritance pattern.

				Number of prenatal cases & (%) in each bin					
**Ancestry**	**Case definition**	**Inheritance Pattern**	N	**0-12.5%**	**12.5-37.5%**	**37.5-62.5%**	**62.5-87.5%**	**87.5-100%**	**C-A test Z-statistic**
**African**	Positive	Autosomal dominant de novo	34	33	1	0	0	0	-1.33
		Autosomal dominant inherited	4	4	0	0	0	0	-0.55
		Autosomal dominant unknown	1	1	0	0	0	0	-0.27
		Autosomal recessive (compound heterozygous)	11	11	0	0	0	0	-0.90
		Autosomal recessive (homozygous)	4	4	0	0	0	0	-0.55
		X-linked	6	6	0	0	0	0	-0.67
	Inconclusive	Autosomal dominant de novo	6	3	3	0	0	0	1.12
		Autosomal dominant inherited	4	3	1	0	0	0	0.19
		Autosomal dominant unknown	1	1	0	0	0	0	-0.27
		Autosomal recessive (compound heterozygous)	3	3	0	0	0	0	0.19
		Autosomal recessive (homozygous)	3	3	0	0	0	0	0.19
		X-linked	2	2	0	0	0	0	-0.39
	Negative	Negative	237	216	9	4	5	3	
**Native American**	Positive	Autosomal dominant de novo	34	24	5	5	0	0	0.22
		Autosomal dominant inherited	4	2	1	0	1	0	1.43
		Autosomal dominant unknown	1	1	0	0	0	0	-0.51
		Autosomal recessive (compound heterozygous)	11	10	1	0	0	0	-1.30
		Autosomal recessive (homozygous)	4	2	0	2	0	0	1.43
		X-linked	6	5	0	0	1	0	0.27
	Inconclusive	Autosomal dominant de novo	6	5	0	1	0	0	-0.23
		Autosomal dominant inherited	4	3	1	0	0	0	-0.39
		Autosomal dominant unknown	1	0	0	1	0	0	1.95
		Autosomal recessive (compound heterozygous)	3	3	0	0	0	0	-0.87
		Autosomal recessive (homozygous)	3	2	0	0	1	0	1.24
		X-linked	2	2	0	0	0	0	-0.71

	Negative	Negative	237	179	28	22	7	1	
**East Asian**	Positive	Autosomal dominant de novo	34	27	1	3	0	3	0.45
		Autosomal dominant inherited	4	4	0	0	0	0	-0.78
		Autosomal dominant unknown	1	1	0	0	0	0	-0.39
		Autosomal recessive (compound heterozygous)	11	9	0	0	0	2	0.72
		Autosomal recessive (homozygous)	4	4	0	0	0	0	-0.78
		X-linked	6	5	0	1	0	0	-0.26
	Inconclusive	Autosomal dominant de novo	6	2	1	1	1	1	2.42
		Autosomal dominant inherited	4	4	0	0	0	0	-0.78
		Autosomal dominant unknown	1	1	0	0	0	0	-0.39
		Autosomal recessive (compound heterozygous)	3	2	0	1	0	0	0.30
		Autosomal recessive (homozygous)	3	3	0	0	0	0	-0.67
		X-linked	2	0	0	0	0	2	4.09
	Negative	Negative	237	202	3	10	2	20	
**European**	Positive	Autosomal dominant de novo	34	6	4	6	7	11	0.32
		Autosomal dominant inherited	4	0	1	0	1	2	0.95
		Autosomal dominant unknown	1	0	0	0	0	1	1.16
		Autosomal recessive (compound heterozygous)	11	3	0	0	3	5	0.75
		Autosomal recessive (homozygous)	4	2	2	0	0	0	-2.41
		X-linked	6	1	0	1	1	3	0.88
	Inconclusive	Autosomal dominant de novo	6	1	3	2	0	0	-1.86
		Autosomal dominant inherited	4	1	0	0	2	1	0.28
		Autosomal dominant unknown	1	0	0	1	0	0	-0.20
		Autosomal recessive (compound heterozygous)	3	0	1	0	0	2	0.82
		Autosomal recessive (homozygous)	3	1	1	1	0	0	-1.51
		X-linked	2	2	0	0	0	0	-2.18
	Negative	Negative	237	41	40	33	54	69	
**Middle Eastern**	Positive	Autosomal dominant de novo	34	25	8	1	0	0	0.23
		Autosomal dominant inherited	4	3	1	0	0	0	-0.07

		Autosomal dominant unknown	1	1	0	0	0	0	-0.47
		Autosomal recessive (compound heterozygous)	11	9	2	0	0	0	-0.50
		Autosomal recessive (homozygous)	4	2	1	0	0	1	3.12
		X-linked	6	5	1	0	0	0	-0.44
	Inconclusive	Autosomal dominant de novo	6	6	0	0	0	0	-1.14
		Autosomal dominant inherited	4	4	0	0	0	0	-0.94
		Autosomal dominant unknown	1	0	1	0	0	0	1.26
		Autosomal recessive (compound heterozygous)	3	3	0	0	0	0	-0.81
		Autosomal recessive (homozygous)	3	2	0	0	0	1	2.94
		X-linked	2	2	0	0	0	0	-0.66
	Negative	Negative	237	183	48	4	0	2	
**South Asian**	Positive	Autosomal dominant de novo	34	31	0	2	0	1	-0.13
		Autosomal dominant inherited	4	4	0	0	0	0	-0.54
		Autosomal dominant unknown	1	1	0	0	0	0	-0.27
		Autosomal recessive (compound heterozygous)	11	10	0	0	0	1	0.36
		Autosomal recessive (homozygous)	4	3	0	0	0	1	1.50
		X-linked	6	6	0	0	0	0	-0.66
	Inconclusive	Autosomal dominant de novo	6	6	0	0	0	0	-0.66
		Autosomal dominant inherited	4	3	0	0	0	1	1.50
		Autosomal dominant unknown	1	1	0	0	0	0	-0.27
		Autosomal recessive (compound heterozygous)	3	3	0	0	0	0	-0.47
		Autosomal recessive (homozygous)	3	2	0	1	0	0	0.74
		X-linked	2	2	0	0	0	0	-0.38
	Negative	Negative	237	220	1	2	0	14	

Supplementary Table 4: Recurrent diagnostic pathogenic mutations found among pediatric and prenatal cases.

**Gene**	**Nucleotide change and amino acid substitution**	**Inheritance pattern**	**Pediatric/ Prenatal**	**Race/Ethnicity categories reported by parents of case 1**	**Race/Ethnicity categories reported by parents of case 2**
PTPN11	c.182A>G<br><br>p.Asp61Gly	Autosomal dominant de novo	Pediatric, prenatal.	LT	Missing
CDK13	c.2525A>G<br><br>p.Asn842Ser	Autosomal dominant de novo	Both pediatric	LT	EU, LT, NAT
FGFR3	c.742C>T<br><br>p.Arg248Cys	Autosomal dominant de novo, Autosomal dominant unknown (father unavailable)	Both prenatal	EU	EA, EU, Missing
PTPN11	c.854T>C<br><br>p.Phe285Ser	Autosomal dominant de novo	Both prenatal	Missing	EU

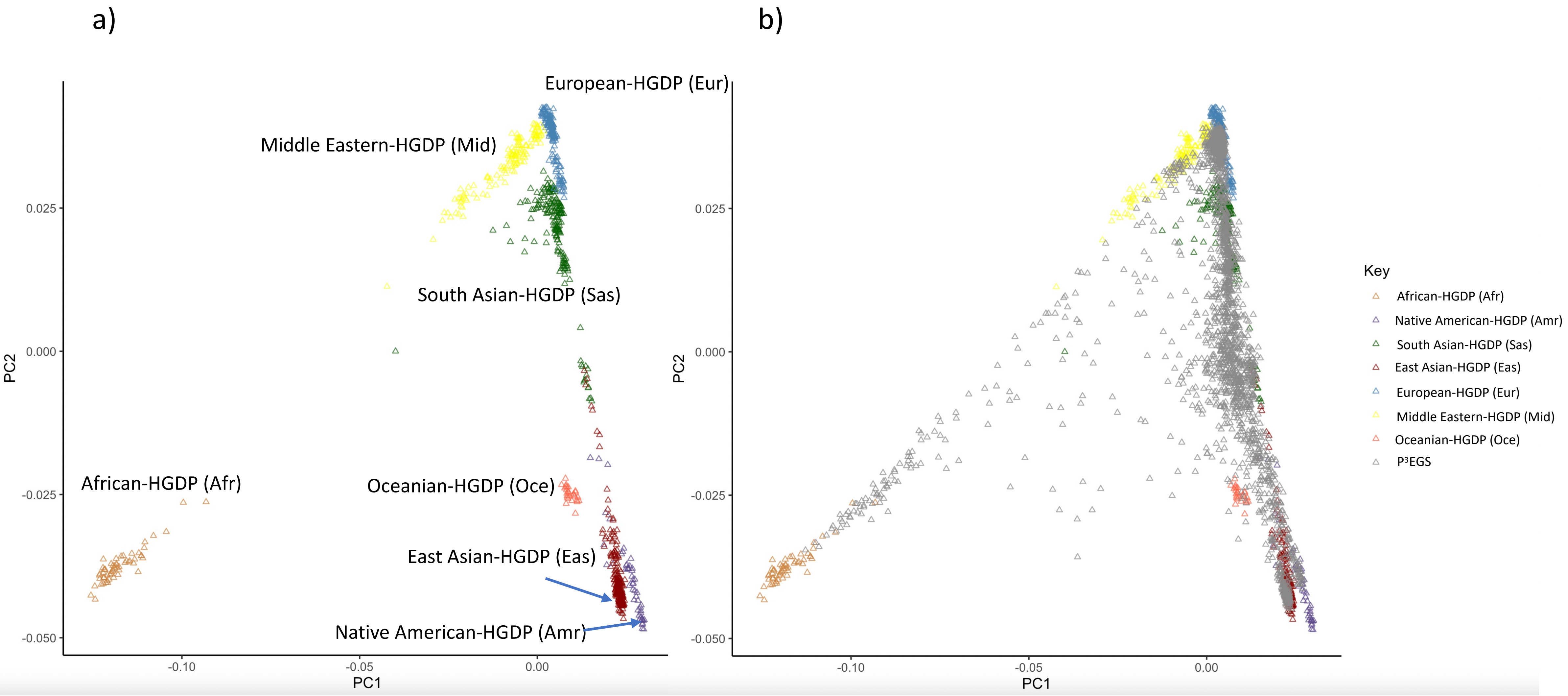

Supplementary Figure 1: PC 1 and PC 2 HGDP samples and P^{3}EGS participants projected; a) only HGDP samples; b) HGDP samples and P^{3}EGS participants

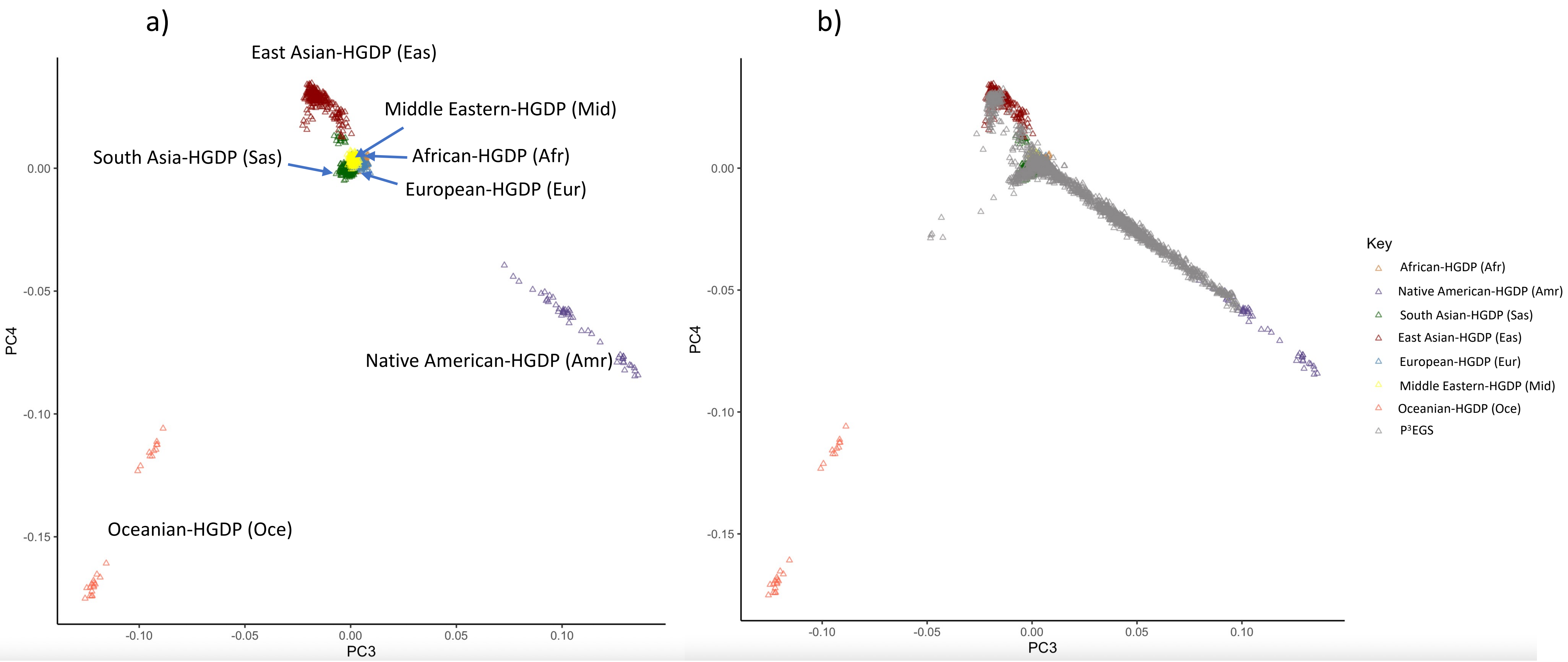

Supplementary Figure 2: PC 3 and PC 4 HGDP samples and P[3]EGS participants projected; a) only HGDP samples; b) HGDP samples and P[3]EGS participants

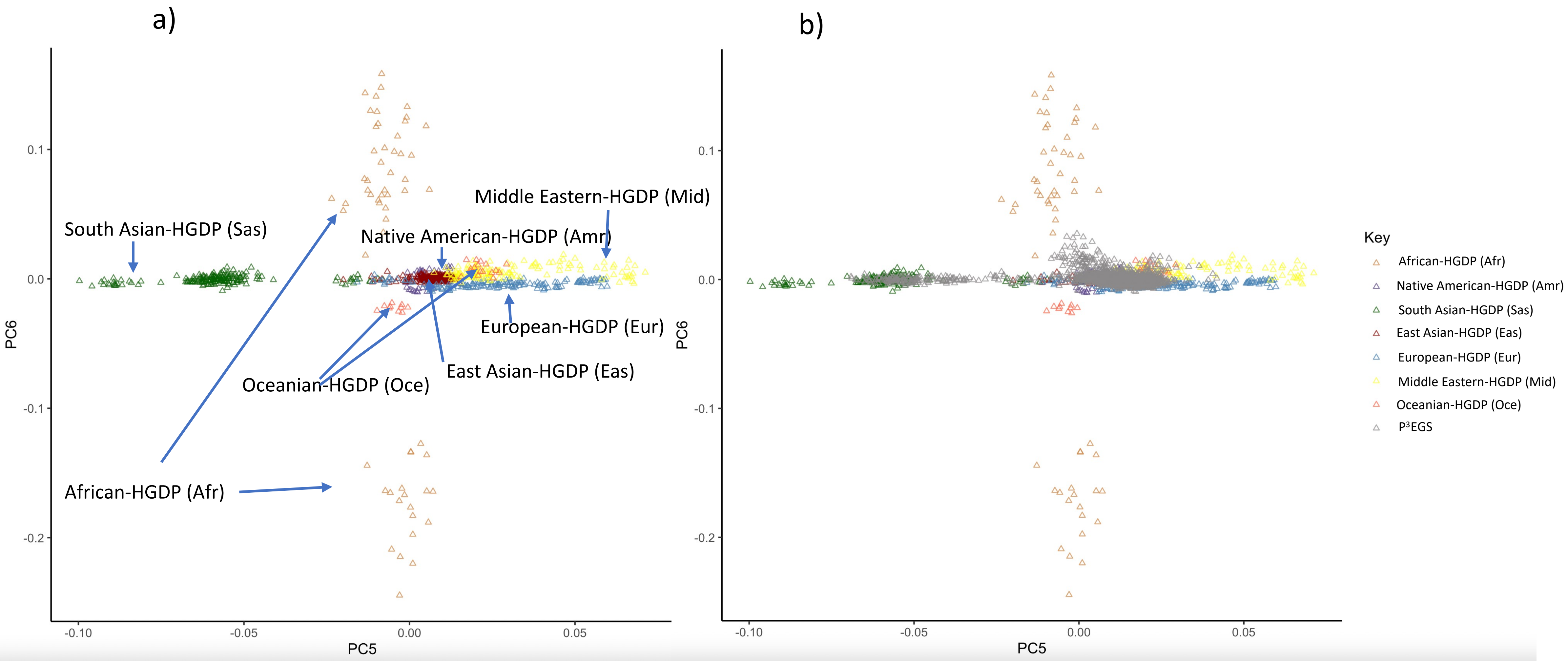

Supplementary Figure 3: PC 5 and PC 6 HGDP samples and P^{3}EGS participants projected; a) only HGDP samples; b) HGDP samples and P^{3}EGS participants

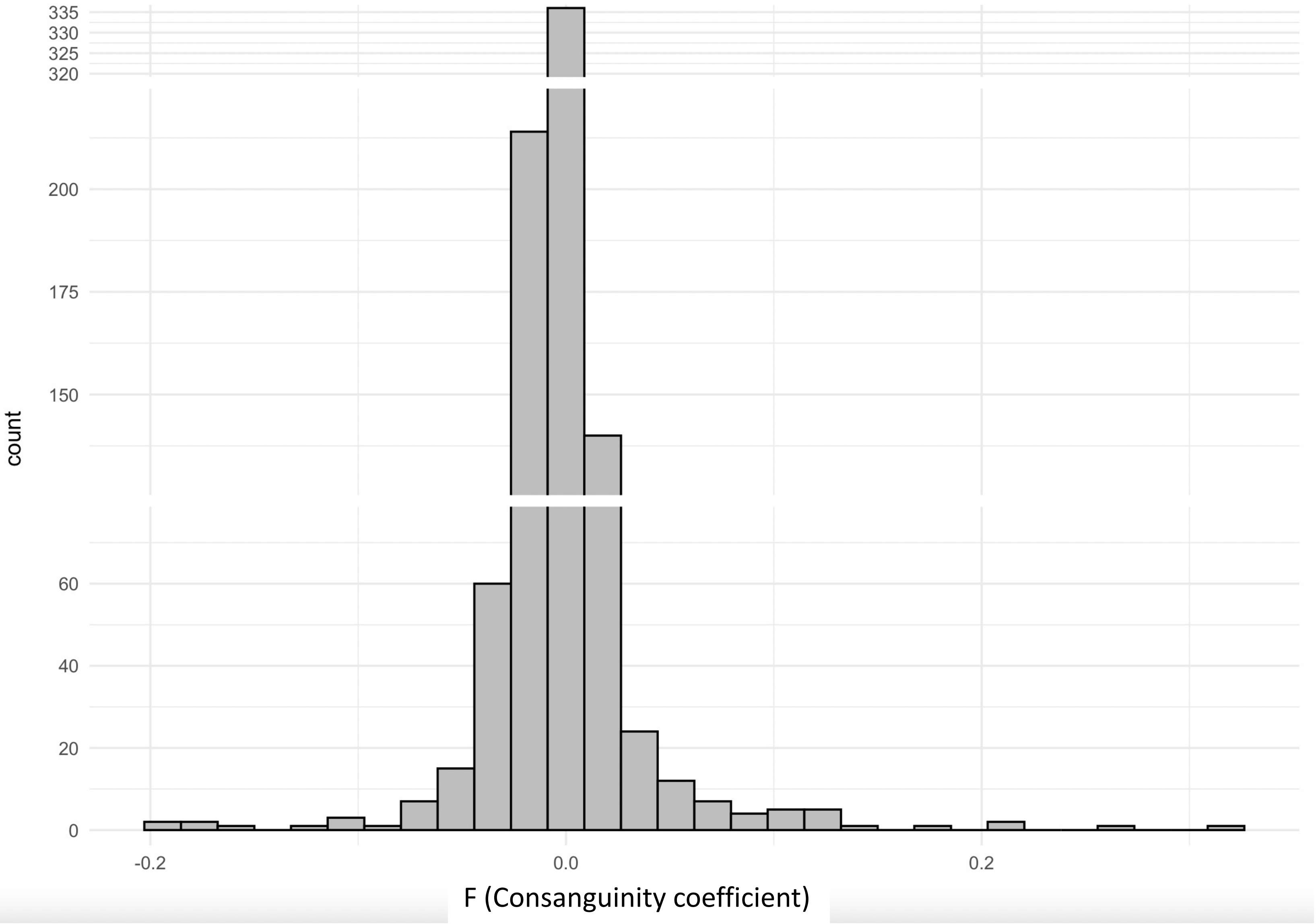

Supplementary Figure 4: Histogram of Fs in P³EGS pediatric and prenatal patients
